# Urban microbial ecology of a freshwater estuary of Lake Michigan

**DOI:** 10.12952/journal.elementa.000064

**Published:** 2015-07-29

**Authors:** Jenny C. Fisher, Ryan J. Newton, Deborah K. Dila, Sandra L. McLellan

**Affiliations:** 1 School of Freshwater Sciences, University of Wisconsin-Milwaukee, Milwaukee, Wisconsin, United States

## Abstract

Freshwater estuaries throughout the Great Lakes region receive stormwater runoff and riverine inputs from heavily urbanized population centers. While human and animal feces contained in this runoff are often the focus of source tracking investigations, non-fecal bacterial loads from soil, aerosols, urban infrastructure, and other sources are also transported to estuaries and lakes. We quantified and characterized this non-fecal urban microbial component using bacterial 16S rRNA gene sequences from sewage, stormwater, rivers, harbor/estuary, and the lake surrounding Milwaukee, WI, USA. Bacterial communities from each of these environments had a distinctive composition, but some community members were shared among environments. We used a statistical biomarker discovery tool to identify the components of the microbial community that were most strongly associated with stormwater and sewage to describe an “urban microbial signature,” and measured the presence and relative abundance of these organisms in the rivers, estuary, and lake. This urban signature increased in magnitude in the estuary and harbor with increasing rainfall levels, and was more apparent in lake samples with closest proximity to the Milwaukee estuary. The dominant bacterial taxa in the urban signature were *Acinetobacter, Aeromonas*, and *Pseudomonas*, which are organisms associated with pipe infrastructure and soil and not typically found in pelagic freshwater environments. These taxa were highly abundant in stormwater and sewage, but sewage also contained a high abundance of *Arcobacter* and *Trichococcus* that appeared in lower abundance in stormwater outfalls and in trace amounts in aquatic environments. Urban signature organisms comprised 1.7% of estuary and harbor communities under baseflow conditions, 3.5% after rain, and >10% after a combined sewer overflow. With predicted increases in urbanization across the Great Lakes, further alteration of freshwater communities is likely to occur with potential long term impacts on the function of estuarine and nearshore ecosystems.

## Introduction

Urban ecology addresses the interactions of organisms and the environment within built landscapes (Grimm et al., 2008). Although urban ecology has developed into a multi-disciplinary field including biological, physical, social, and built components, the urban *microbial* ecology of these complex systems has yet to be fully integrated into such studies. Bacteria can provide essential ecosystem services such as nutrient cycling (Fenchel et al., 2012) and pollutant degradation (Chaudhry and Chapalamadugu, 1991; Samanta et al., 2002; Singh and Walker, 2006), but their community composition and function in urban aquatic environments are poorly understood. Urban reservoirs of bacteria include aerosols (Brodie et al., 2007), soil and vegetation (Kaye et al., 2005), microbiomes associated with humans and other macroorganisms (Cao et al., 2011), as well as the built environment of roads, buildings, and pipes (Schiff and Kinney, 2001; VandeWalle et al., 2012; King, 2014). Stormwater can serve as a conduit for delivering urban-derived microorganisms to aquatic environments, but little is known about the bacterial communities in stormwater or the fate of these organisms in the environment (Wenger et al., 2009). Biological monitoring of stormwater has traditionally focused on identifying fecal indicator bacteria (Jeng et al., 2005; Mallin et al., 2009; Parker et al., 2010; Sauer et al., 2011; Sercu et al., 2011; Sidhu et al., 2012), which serve as sentinels for human pathogens and breaches in sewer lines. While some studies have considered the impact of stormwater bacterial communities in streams and basins receiving runoff (Mallin et al., 2009; Sercu et al., 2009; Badin et al., 2011; Wang et al., 2011), few have characterized non-fecal constituents of the bacterial communities of urban runoff directly from storm drains and outfalls (Wu et al., 2010). Thus stormwater input remains a black box in terms of tracking the flux of urban-associated bacteria through watersheds and into coastal environments.

Freshwater estuaries are often centers of intense urban development because of the abundant ecosystem services provided, from fisheries to recreation to assimilation of urban waste (Larson et al., 2013). These estuaries are mixing zones where chemically and biologically distinct river and urban runoff inputs meet the lake, bringing with them a mélange of nutrients, pollutants, sediment, and bacteria (Stephens and Minor, 2010; Howell et al., 2012). Rivers and streams that terminate in the Great Lakes drain several densely populated urban centers in addition to upstream agricultural watersheds (Larson et al., 2013). Development and urbanization over the past century have increased the proportion of impervious surfaces (Arnold and Gibbons, 1996), thereby exacerbating the impacts of stormwater by increasing the volume of runoff and loads of non-natural inputs (chemicals, metals, trash) (Paul and Meyer, 2001). The microbial component of urban pollution to Lake Michigan has been addressed in relation to recreational water impairment and human health impacts, as many of the Great Lakes beaches experience multiple closings and swimming advisories (McLellan and Salmore, 2003; Alm et al., 2006; Wong et al., 2009; Ge et al., 2012). In addition to shoreline contamination, human fecal markers have been found consistently in the Milwaukee estuary (Bower et al., 2005; McLellan et al., 2007; Newton et al., 2013). These studies illustrate both the extent and duration of the impact from urban runoff and infrastructure failure from leaking sewage pipes or combined sewer overflows (CSOs). However, studies have yet to address the ecological impacts of non-fecal bacteria from the urban environment when they are transported to the estuary and lake.

Here we examine the microbial composition of stormwater and sanitary sewage and track the presence and relative abundance of organisms associated with urban infrastructure and runoff into the Milwaukee estuary and harbor under baseflow versus moderate to extreme rainfall conditions. We use deep sequencing to assess the proportion and identity of the coastal bacterial community that comes from urban sources – in particular, stormwater runoff and sanitary and storm sewer infrastructure (pipes). Based on the fate and persistence of urban-derived bacteria to aquatic systems, we consider potential ecological implications for the presence of these organisms in the estuarine environment.

## Methods

### Study site and sampling

**Figure 1:**
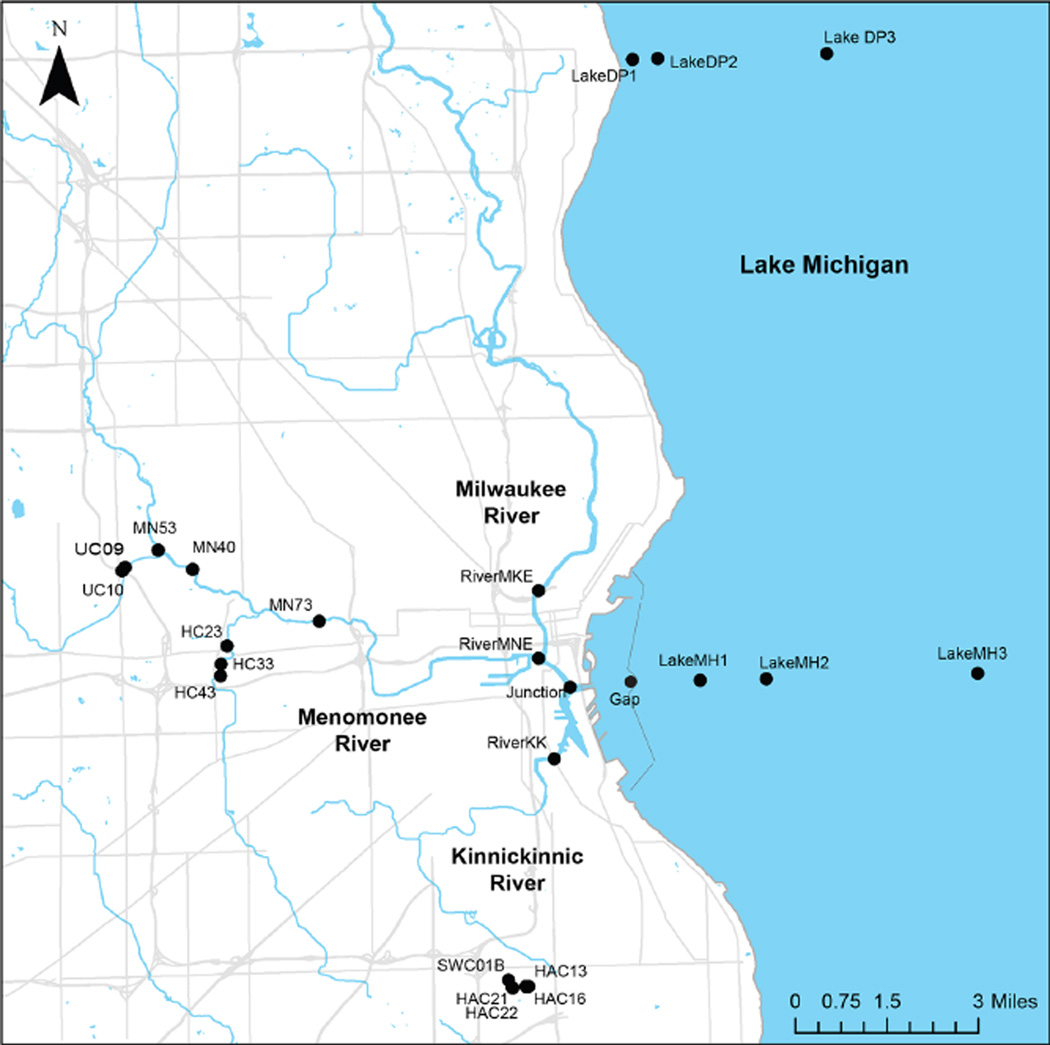
Map of sampling sites from Milwaukee stormwater outfalls, rivers, harbor and Lake Michigan Our study area included stormwater outfalls and pipes, and surface water sites on three rivers within metropolitan Milwaukee and in Lake Michigan. The Kinnickinnic River, Menomonee River, and Milwaukee River converge in the Milwaukee estuary (Junction) just prior to discharging to Lake Michigan through the main opening in the harbor breakwall (Gap). A total of six lake sites were sampled offshore from the Milwaukee Harbor and Doctor’s Park. Stormwater outfall samples were collected from Holmes Avenue Creek, Wilson Park Creek, Honey Creek, Underwood Creek, and Menomonee River. doi: 10.12952/journal.elementa.000064.f001

Our study area included stormwater outfalls and pipes, surface water sites on three rivers within metropolitan Milwaukee, the Milwaukee estuary and harbor, and nearshore Lake Michigan (Figure 1). The Kinnickinnic River (designated RiverKK) and the Menomonee River (RiverMNE) drain highly urbanized watersheds, while the Milwaukee River (RiverMKE) drains a large, mixed land use watershed. The three rivers converge in an estuary (Junction) just prior to discharging directly to Lake Michigan through the main opening in the harbor breakwall (Gap). Breakwalls surrounding the Milwaukee Harbor limit the exchange of water between the estuary and lake to the north and south, with the majority of transport occurring directly east through the Gap site. Lake sites extended to 8 km offshore from the Milwaukee Harbor (LakeMH) and 15 km north of the harbor to Doctor’s Park (LakeDP). Stormwater outfall samples are named based on the tributary to which they discharge: HAC = Holmes Avenue Creek, WC = Wilson Park Creek, HC = Honey Creek, UC = Underwood Creek, MN = Menomonee River; the one inline sample (SWC01B) is preceded by an “S.” The water samples collected from infrastructure (stormwater outfalls and pipes, sewage influent) and aquatic environments (rivers, estuary/harbor, lake) represent the sources and sinks for urban bacteria in this study. Lake samples from outside the harbor/estuary were collected only during baseflow conditions and serve as the non-impacted end member in the transect. Stormwater samples collected from outfalls and pipes throughout Milwaukee represent a range of rain intensity and land use conditions; river and estuary/harbor samples reflect dry and wet weather and a combined sewage overflow (CSO) event.

During spring and summer of 2010–2012, we collected stormwater samples from terminal outfalls and one inline stormwater pipe during rain events of varying intensity (Table 1). Table S1 provides additional sample metadata, including rainfall amounts and sequencing IDs. Grab samples were collected directly from the outfall flows; 500 mL bottles were rinsed three times with sample prior to final collection. An automated sampler (Teledyne ISCO, Lincoln, NE) controlled by a flow sensor collected the inline sample. River samples were collected as surface water grab samples in rinsed buckets into 1 L plastic bottles (rinsed three times with sample prior to final collection). All samples were stored on ice during transport to the lab. Two transects of lake samples were taken: one from Doctor’s Park (non-urban) and one directly east through the Milwaukee Harbor (urban) during baseflow conditions, where there was no rainfall in the past 48 hours. Multiple samples were taken in the estuary (Junction and Gap) to capture baseflow, rain, and CSO conditions. All lake water samples were surface water grab samples from surface to ~0.5 m depth, collected into 2 or 4 L bottles, stored on ice, and returned to the lab for filtering within 4 hours of sampling. Lake samples were collected during baseflow and represent the non-impacted aquatic end member. Sewage samples were flow-weighted composite samples of primary influent sewage collected over a 24-hour period from the two Milwaukee wastewater treatment plants, Jones Island Water Reclamation Facility and South Shore Water Reclamation Facility.

### DNA extraction and sequencing

For DNA extraction, water from each sample type (sewage: 25 mL, stormwater: 200 mL, river: 200–400 mL, harbor and lake: 400 mL) was filtered onto a 0.22 µm pore size nitrocellulose filter (47 mm diameter; Millipore, Billerica, MA) and stored at −80 °C prior to extraction procedure. The frozen filters were broken into small fragments using a sterile metal spatula. DNA was extracted using the MPBIO FastDNA® SPIN Kit for Soil (MP Biomedicals, Santa Anna, CA) according to manufacturers instructions, with the exception of the lysis step in which a bead beater (BioSpec, Bartlesville, OK) was used for 2 minutes. DNA was eluted in the final step using 150 µl of DES.

We selected stormwater samples previously shown (McLellan and Dila, 2014) to have low or no evidence of human fecal contamination based on quantitative PCR (qPCR) screening using two molecular markers for human sewage including human *Bacteroides* and human *Lachnospiraceae* (Bower et al., 2005; Newton et al., 2011b). We included one stormwater sample (MN73) with high copy numbers of both human fecal markers to compare to clean samples in our analyses.

Amplicon sequencing of the V6 hypervariable region of the 16S rRNA gene from bacteria was conducted at the Josephine Bay Paul Center at the Marine Biological Laboratory (Woods Hole, MA). Sequencing employed five fusion primers at the 5′ end of the region and four primers at the 3′ end to amplify a total of ~100 bp (*E. coli* positions 967–1064) including primers. Briefly, V6 amplicons were generated in three separate PCR reactions of 25 cycles plus 5 cycles of PCR with custom fusion primers. Pooled samples were sequenced using v3 chemistry on an Illumina HiSeq 1000 (Eren et al., 2013). Bioinformatic quality filtering reduced the frequency of sequencing-based errors by removing any mismatches between the forward and reverse reads (Eren et al., 2013).

### Data analysis and statistics

We used the *sub.sample* function in mothur v. 1.24 (Schloss et al., 2009) to generate evenly sized sequence datasets equal to the abundance of the smallest sample (686,833 reads). Prior to subsampling, sequence reads ranged from 760,837 to 1,346,570 with a median of 987,809 reads. Taxonomic assignments generated by Global Alignment for Sequence Taxonomy (GAST) (Huse et al., 2008) were downloaded from VAMPS (vamps.mbl.edu) (Huse et al., 2014) and were used for all taxonomy-based analyses.

We conducted all data analyses in the statistical package R (R Development Core Team, 2012); the *vegan* package (Oksanen et al., 2013) provided tools for community analysis. Non-metric multidimensional scaling (NMDS) analysis and hierarchical clustering were based on Bray-Curtis dissimilarities and used taxonomic count data as input. Data were transformed using Wisconsin double standardization. We compared the taxon abundance within and among environments using *adonis* with the Bray-Curtis dissimilarity index and 999 permutations and examined dispersion effects using *betadisper*. Heatmaps were constructed with the *pheatmap* function using scaled taxonomic counts. Diversity measures (Hill numbers) were calculated with the *renyi* function for individual samples and *renyiaccum* function for average and pooled samples. Venn diagrams were made with the *venn* function in *gplots*. We generated all data visualizations with *ggplot2, gplots*, or base graphics in R.

### Biomarker signature analysis (LEfSe)

We used a statistical biomarker tool that uses linear discriminant analysis effect size (LEfSe) to identify taxa that are preferentially abundant in one type of environment (Segata et al., 2011). LEfSE v1.0 was run via the Galaxy server (http://huttenhower.sph.harvard.edu/galaxy/) with default parameters (bootstrap iterations of 30 and minimum effect size of 2.0). Taxonomic count data were used as input. LEfSE identified taxa that best explained the differences in two or more sets of classes (in this case, sample environment). We performed the analysis with both broad classes (urban versus aquatic) and specific environments (sewage, stormwater, river, harbor, and lake) as classes. LEfSE determined a “biomarker signature” composed of taxa that associated preferentially with a given class. Biomarker taxa from one class could also be present in other classes, but were found in lower abundance or inconsistently.

### Minimum entropy decomposition

Our taxa-based urban signature provided a general identification of potential bacterial sources to the aquatic environment from our highly diverse stormwater samples. This approach allowed us to observe overall trends in urban signature; however, ecotypes within taxonomic groups may be specific to either the urban or aquatic environment. To more accurately track specific organisms from urban to aquatic environments, we refined the urban-associated taxa identified by LEfSE analysis into DNA sequence-based operational taxonomic units (OTUs). As we were interested in bacteria from runoff and urban *infrastructure*, we excluded sequences that were classified to the major human fecal taxa found in sanitary sewage: *Lachnospiraceae, Ruminococcaceae, Bacteroidaceae, Porphyromonadaceae*, and *Rikenellaceae* (Newton et al., 2015). We further refined the urban signature to include only the taxa with a minimum mean relative abundance of 0.1% in urban samples.

The minimum entropy decomposition (MED) pipeline version 1.2 (Eren et al., 2015) assigned DNA sequences associated with urban taxa into OTUs. MED is a method for creating OTUs that differs from traditional methods, which use a fixed percent similarity threshold (usually 97%) to group sequences. MED instead uses nucleotide entropy along the length of the DNA sequences to discern relevant differences in nucleotides (genetic variation) from noise (sequence error). The total pool of sequences from all samples is partitioned into sequentially refined “nodes” based on the nucleotide present in the position with the highest entropy (greatest amount of variability). This iterative process continues until nodes achieve a minimum entropy threshold. The MED algorithm provides superior grouping in terms of taxonomic homogeneity over traditional OTU-picking procedures, particularly with short reads produced by high-throughput sequencing (Eren et al., 2015).

We set the minimum substantive abundance criterion (*M*) to 86, which is equivalent to 0.001% of the total sequences that mapped to urban signature taxa, to reduce noise from rare sequences. We also allowed within-node variation (*V*) of 2 nucleotides and used default criteria for all other parameters. Of the 8,584,339 sequences used in the analysis, the MED algorithm discarded 168,029 sequences that failed the minimum substantive abundance criterion and 64,207 that did not meet the within-node variance criterion.

### Sequence archive

Sequencing files for stormwater, river, harbor and lake sequences are publicly available in the National Center for Biotechnology Information (NCBI) Sequence Read Archive under accession number SRP056973; sewage samples are in project SRP041262.

## Results

### Microbial communities of urban sources and environmental sinks

**Figure 2:**
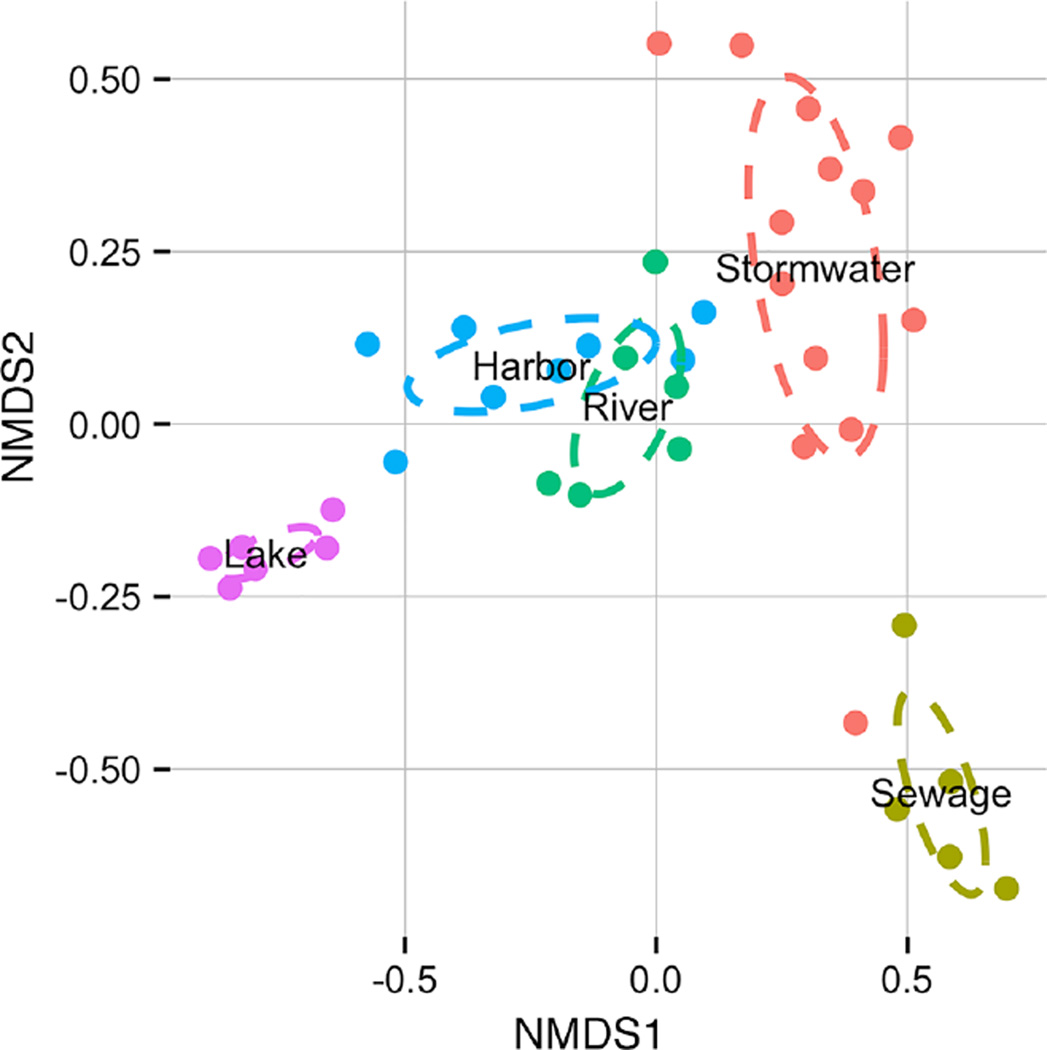
Ordination of sample sites based on bacterial taxonomic composition A non-metric multidimensional scaling plot based on Bray-Curtis dissimilarity of bacterial taxonomic composition across samples is illustrated. The run stress was 0.10, indicating a good fit. Ellipses indicate the dispersion of group based on a weighted covariance matrix of samples distance from the centroid. Individual samples are colored by environment: stormwater=coral, sewage=yellow-green, rivers=green, harbor=blue, lake=purple. doi: 10.12952/journal.elementa.000064.f002

High throughput sequencing allowed us to deeply characterize the microbial communities from sanitary sewage, stormwater, rivers, estuary/harbor, and nearshore Lake Michigan off the coast of Milwaukee, WI, USA. Analysis of samples based on taxonomic classification revealed consistent community patterns associated with both urban and aquatic environments. Individual samples from the same type of environment formed distinct clusters in a NMDS ordination of taxa presence and relative abundance (Figure 2). Only the harbor and river groups shared overlap of covariance ellipses; and a single stormwater sample, previously shown to have a high abundance of human fecal indicators, clustered with sewage rather than stormwater. Sewage grouped most closely with stormwater, while the aquatic environments were ordered from rivers to estuary to lake with increasing distinction from the urban sources. Bacterial communities within each of the five environments tended to cluster together, and variability among environments was higher than within-group variability (*adonis* F-ratio = 14.1, R^2^= 0.63, p = 0.001). Group variance, as determined by average distance to centroid, was greatest in stormwater (0.42) and lowest in lake samples (0.070), with similar dispersion within sewage (0.23), river (0.24), and harbor (0.21) samples.

**Figure 3:**
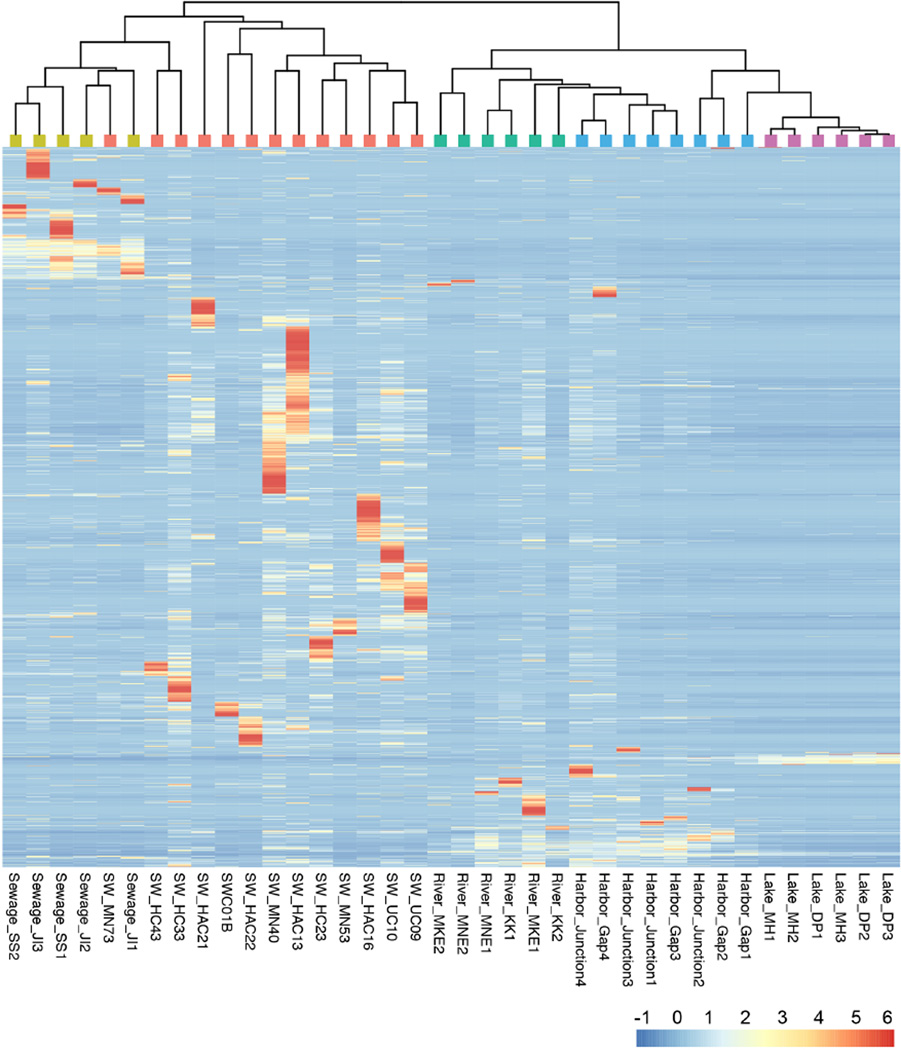
Heat map of taxon relative abundance and hierarchical clustering of samples Taxon relative abundance is represented by the heatmap. Values are scaled by taxon relative abundance across all samples: red indicates a taxon with skewed distribution, with the presence of a taxon concentrated in one or two samples; white indicates an even distribution among samples; and blue represents lower relative abundance or absence. Both the taxa and samples were clustered using Bray-Curtis dissimilarities. The colors on the cluster dendrogram correspond to the environments in the NMDS plot in Fig 2: coral = stormwater, yellow-green=sewage, green=rivers, blue=harbor, purple=lake. doi: 10.12952/journal.elementa.000064.f003

We examined the distribution of bacterial taxa among the five groups (sewage, stormwater, river, harbor and lake) to identify both the general composition of each environment as well as taxa preferentially associated with either urban or aquatic sources. Taxa distributions in individual samples are shown as a heatmap (Figure 3). Hierarchical clustering of samples and taxa within those samples reveals patterns associated with each environment and facilitates visualization of the >2800 taxa. Similar to the NMDS analysis, samples from a particular environment grouped together, with the exception of the human fecal contaminated stormwater sample, which closely resembled sewage (SW_MN73). While certain taxa were associated almost exclusively with individual samples (shown as the dark red zones), many taxa were found in most or all samples from the same environment, and some taxa had a cosmopolitan distribution. Many of the taxa from stormwater also appeared in river and harbor samples, while the lake samples had an even distribution of only a few taxa. A detailed analysis of individual and group sample diversity confirmed the patterns shown in the heatmap (Table S2). Stormwater samples as a group, as well as for individual members, had the highest diversity in terms of taxon richness, while the lake community and its constituents had the lowest group and individual taxon richness. Sewage, rivers and harbors shared similar diversity levels; all environments were highly uneven, as characterized by the presence of a few highly abundant taxa and many rare taxa.

### Urban and aquatic signatures (LEfSe Analysis)

**Figure 4:**
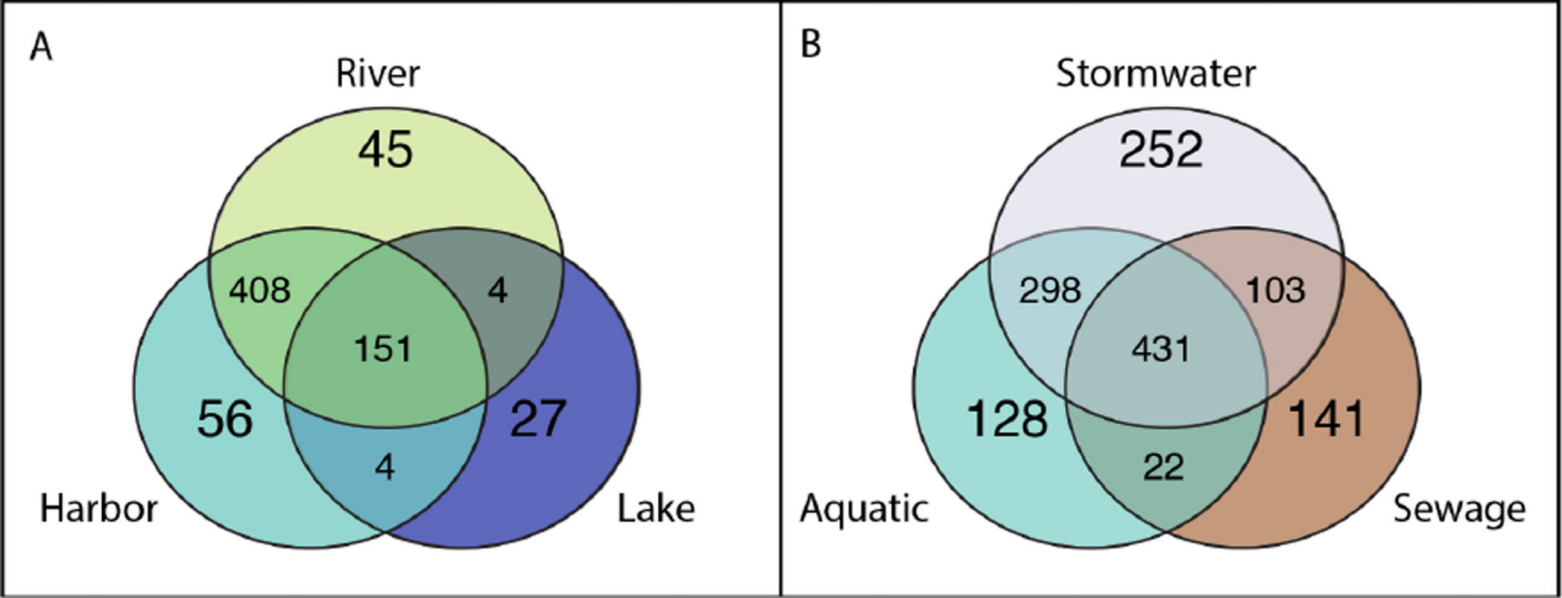
Venn diagram of biomarker taxa and cosmopolitan taxa from urban and aquatic environments Venn diagrams show the distribution of biomarker signature taxa, and rare or nonpreferentially distributed taxa among A) rivers, harbor, and lake and among B) aquatic sources, sewage, and stormwater. The number of taxa identified by LEfSe analysis as biomarkers are shown as associated with their particular environment, although they may also be associated in lower abundance in other environments. The majority of taxa observed in the study had a cosmopolitan distribution, but low abundance in both urban and aquatic environments. doi: 10.12952/journal.elementa.000064.f004

Understanding the characteristic community structure of a given environment, in terms of diversity, evenness, and taxonomic composition, is important for establishing a baseline for the native community so that contributions from outside sources can be identified. We used the biomarker discovery tool LEfSE to identify the taxa that were differentially associated with each environment versus taxa that might be present in an environment but transported from another. For example, leaking sewage infrastructure could imprint a “sewage signature” in stormwater, as was observed for stormwater sample SW_MN73. Similarly, the estuary is a mixing zone that should contain organisms from both the lake and rivers. LEfSe identified preferentially distributed taxa based on their relative abundance in one environment compared to others. LEfSe showed that the three aquatic environments each had several distinguishing biomarker taxa—lake: 27 taxa, harbor: 56 taxa, and rivers: 45 taxa—but many more taxa that were more evenly distributed between the lake, harbor, and river. Over 150 taxa were cosmopolitan to the three aquatic environments and distributed without a clear preference pattern (Figure 4A). The gradient of shared taxonomic composition in this system illustrated the extensive overlap between the rivers and harbor (408 taxa), and less overlap between rivers, harbor, and lake (151 taxa).

LEfSe revealed more biomarker taxa in stormwater (252) and sewage (141) than for individual aquatic environments, as well as many taxa that were evenly shared between the two urban sources (Figure 4B). A single abundant Actinobacteria was found in all environments, as well as a large number of rare taxa that were found sporadically among samples in both of the urban sources and one or more of the aquatic environments. However, these collectively comprised a relatively small proportion of the total microbial community (~2%). Although the aquatic environments had fewer signature taxa, those taxa were very abundant, demonstrating that these environments have a unique community distinct from the urban sources. The LEfSE results using only two classes (“urban” and “aquatic”) produced similar results in terms of the most abundant signature taxa, but identified fewer overall biomarkers. Table S3 provides the LEfSE results for both analyses.

**Figure 5:**
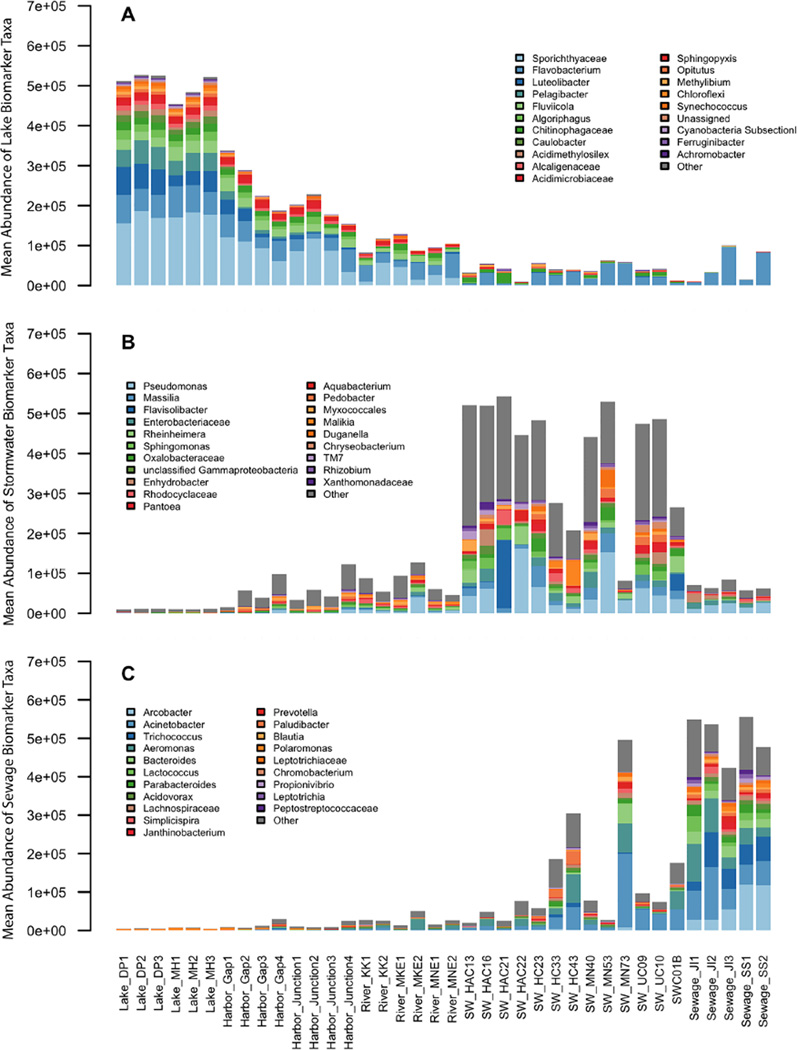
Abundance of biomarker signature taxa The relative abundance of signature taxa from A) lake, B) stormwater, and C) sewage is shown for individual samples. The total sequence reads that mapped to taxa reported as biomarkers from the LEfSe analysis were summed to create a “signature” for each type of environment. The twenty signature taxa with the highest rank abundance are colored uniquely and called out in the legend; the remaining signature taxa are grouped as “other.” doi: 10.12952/journal.elementa.000064.f005

The relative abundance of signature taxa from lake, stormwater, and sewage is shown for individual samples in Figure 5A–C. The signature taxa associated with stormwater and sewage were present in significant abundance in river and harbor samples, with a minimal presence in the lake as well. The 20 most abundant taxa comprised over 90% of the lake signature, while the 20 most abundant stormwater taxa made up only ~50% of the stormwater signature because it was composed of many taxa with lower relative abundance.

The dominant lake signature taxa were unclassified *Sporichthyaceae*, *Luteolibacter, Flavobacterium*, and *Pelagibacter*. Both sewage and stormwater also contained *Flavobacterium*, a lake signature bacterium, but in a lower proportion of the community compared with the lake. *Sporichthyaceae, Luteolibacter*, and *Pelagibacter* were abundant only in aquatic environments. Stormwater signature taxa included *Pseudomonas*, *Flavisolibacter*, *Sphingomonas*, and multiple named genera and unclassified members of the families *Oxalobacteraceae* and *Enterobacteriaceae*. The dominant distinctive taxa in sewage were non-fecal organisms: *Acinetobacter, Aeromonas, Arcobacter*, and *Trichococcus*. Human fecal groups from *Bacteroidales* and *Lachnospiraceae* were present as well, but in significantly lower abundance than the non-fecal organisms. Several of the non-fecal sewage biomarkers such as *Acinetobacter* and *Aeromonas* were also abundant in stormwater, suggesting that these may be organisms that are ubiquitously associated with urban infrastructure.

### Urban signature in aquatic environments

**Figure 6:**
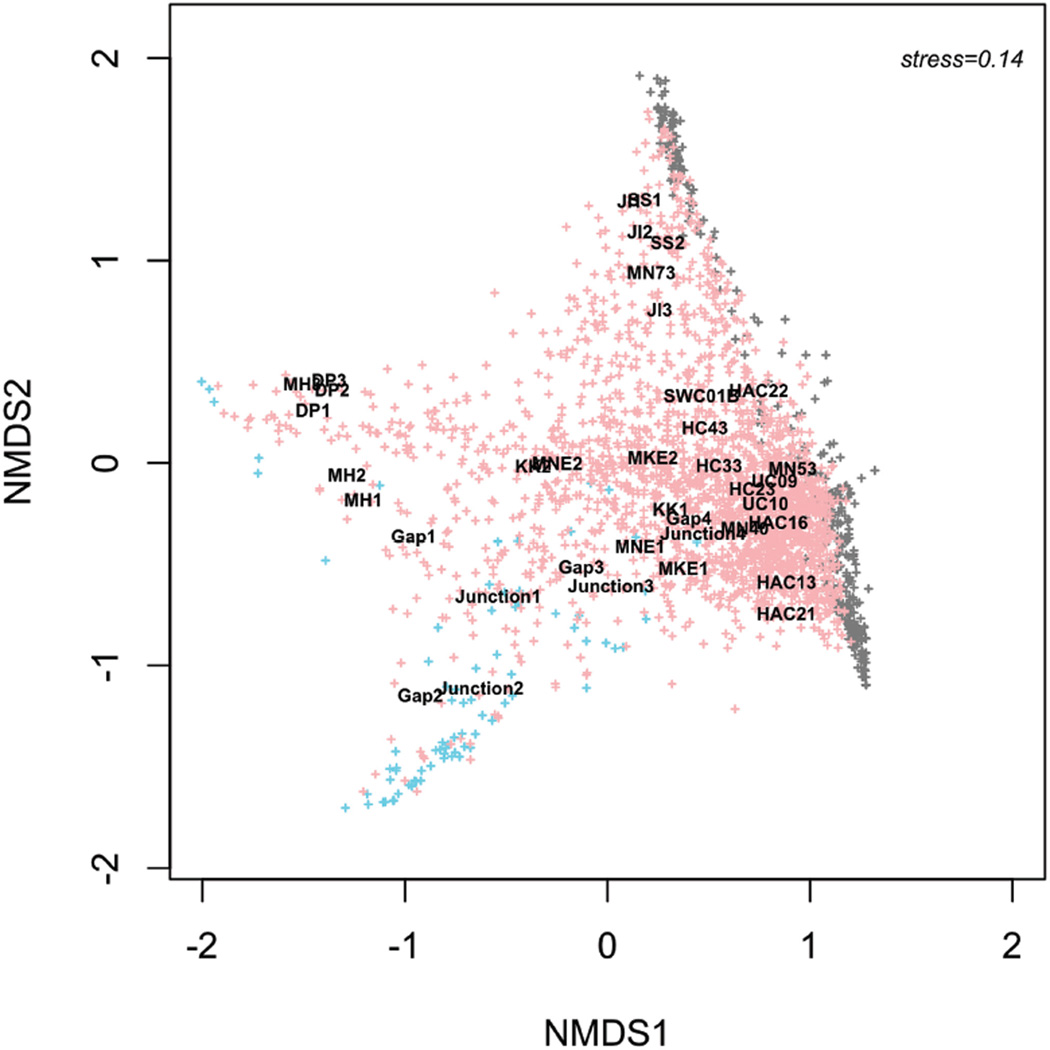
Distribution of OTUs from urban signature taxa and percent urban signature in aquatic environments Sequence-based analysis of 104 taxa within the urban infrastructure signature (stormwater taxa + nonfecal sewage taxa) identified 2,746 OTUs from over 8 million sequences; some OTUs were found exclusively in either urban (427; shown in grey) or aquatic (80; shown in blue) environments, but 2239 OTUs were found in both (pink). NMDS ordination of individual samples based on OTUs is overlaid to show how OTUs distributed in relation to samples and environments. doi: 10.12952/journal.elementa.000064.f006

We used the observable differences in taxonomic composition among environments as the basis for a finer-level sequence-based analysis. For example, the consistent occurrence of a lake signature taxon (*Flavobacterium*) in sewage and stormwater samples, which have no direct input from the lake, suggests that a deeper analysis of the urban signature taxa might reveal organisms within those taxa that are specific to either urban or aquatic environments. We performed a sequence-based analysis of the most prevalent taxa within the urban infrastructure signature (104 stormwater and non-fecal sewage biomarker taxa identified by LEfSe) to better track specific organisms from urban sources into the harbor. MED-based analysis produced 2,746 OTUs from over 8 million sequences; some OTUs were found exclusively in either urban (427) or aquatic (80) environments, but 2239 OTUs were found in both. Figure 6 shows the clustering of the samples based on NMDS of OTUs from urban signature taxa. The association of urban, aquatic, or shared OTUs with different sample clusters allows a simple visualization of how these OTUs distribute in the environment. For example, many sewage OTUs associate solely with sewage, just as some of the OTUs are specific to lake samples or to specific harbor samples.

Two abundant urban signature bacteria from sanitary sewer infrastructure, *Arcobacter* and *Trichococcus*, were dominated by a single OTU in all samples in which they were present. *Aeromonas*, which was dominant in both sewage and stormwater, had two major OTUs that were found in similar proportions in both the urban and aquatic samples. *Acinetobacter* OTUs had varied distributions in sewage and stormwater. One of the two dominant sewage OTUs was in all stormwater, but the other was present in only some stormwater samples; stormwater was more diverse and also had a more even abundance of many *Acinetobacter* OTUs. *Pseudomonas* was also much more diverse and variable in stormwater than sewage—sewage had two major OTUs and a few minor OTUs; stormwater had variable presence and abundance of at least six OTUs with significant abundance. The aquatic samples looked much more similar to stormwater in their *Pseudomonas* OTU distributions.

**Figure 7:**
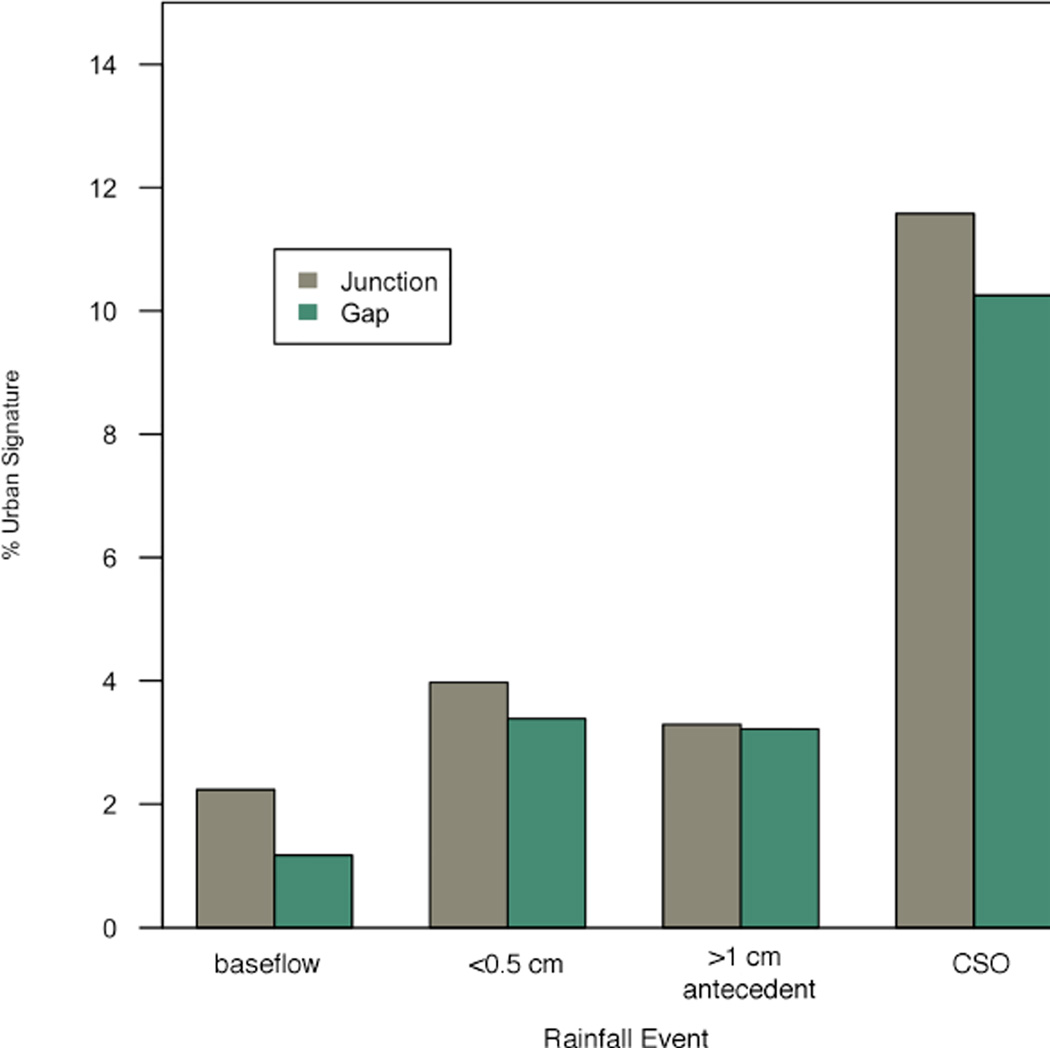
The OTU-based percent urban signature for estuary and harbor samples collected under different weather conditions Junction (estuary) samples are shown in brown; Gap (harbor) samples are shown in green. doi: 10.12952/journal.elementa.000064.f007

The percentage of the shared urban signature OTUs in the estuary and harbor under different rain conditions is shown in Figure 7. Overall the estuary and harbor samples showed a trend of increasing urban signature percentage with increasing rainfall intensity, and a slight decrease with distance from urban sources, as would be expected by dilution. The urban signature percentage in harbor samples was 1.2% in the Gap (outer harbor) and 2.2% in the Junction (estuary) during baseflow conditions. Samples collected after a low rain volume (<0.5 cm) or 24–48 h after a larger rainfall (>1 cm) showed an increase to 3.2–4.0% for Gap and Junction; and after a CSO, 10% of the Gap and 12% of Junction surface water bacterial community was from urban sources. The urban percentage in Junction samples was consistently higher than that of Gap samples taken on the same day. Lake samples, which were all collected under baseflow conditions, still showed traces of the urban signature OTUs (<1%), suggesting either a consistent chronic input or that certain urban organisms (or at least their DNA) persist after they are transported to the estuary and lake environments.

## Discussion

### Composition of the urban bacterial signature

Urban runoff is responsible for 32% of water quality impairment in estuaries across the US (USEPA, 2004). Urban stormwater has been found to deliver not only runoff during rain, but also sanitary sewage from failing infrastructure to surface waters, including during dry weather (Stein and Ackerman, 2007; Sercu et al., 2009; Sauer et al., 2011). Additionally, over 24 billion gallons of combined sewage and stormwater flow into Lake Michigan every year during CSO events (USEPA, 2007). Combined sewage includes large volumes of stormwater runoff, but concerns are typically directed at the sanitary sewage portion of this input and its potential impact on human health. In Milwaukee, CSOs are a fairly rare event, now occurring only twice a year on average, due to infrastructure improvements (SEWRPC, 2008); but CSOs occur much more frequently in other cities with combined sewers (USEPA, 2007). While the bacterial composition of sewage has been described in detail (McLellan et al., 2010; VandeWalle et al., 2012; Shanks et al., 2013), little is known about the bacterial communities associated with stormwater. Nearly all of the stormwater microbiological studies have focused on tracking fecal indicators, which make up only a small fraction the total microbial community (Izbicki et al., 2009). Other studies have focused more specifically on human fecal indicators in stormwater infrastructure (Ahmed et al., 2008; Sercu et al., 2009; Parker et al., 2010; Sauer et al., 2011), indicating sanitary sewage contamination of these systems. One of the only studies to characterize the total bacterial community from stormwater drains assessed sites with significant human and non-human fecal contamination (Wu et al., 2010), which can skew the community to look more like sewage than stormwater.

In the current study we considered two major sources of urban bacteria to the aquatic environment: stormwater and sanitary sewer infrastructure. Both of these systems act as collection basins for the urban environment, with stormwater serving primarily as a collector of “outdoor” organisms and sewage as a collector of “indoor” organisms. Stormwater outfalls are point sources that contain an integrated signal from a variety of urban surfaces (e.g., roads, soil, and pipe infrastructure) and discharge directly to local creeks and rivers. Previous work shows some stormwater systems have significant sewage intrusion (Sercu et al., 2009; Sauer et al., 2011); but for this study, we selected samples without sanitary sewage contamination before identifying potential biomarkers. In our study area, there are more than 200 stormwater outfalls in the urban reaches of the rivers leading to Lake Michigan. The hierarchical clustering demonstrated that stormwater has a very consistent and distinct community, including across seasons and two years. While stormwater contains diversity within its runoff component, the infrastructure signal was highly consistent and distinct. We included one stormwater sample that had evidence of sewage contamination based on two indicators of human fecal pollution (McLellan and Dila, 2014); in this sample, the total taxonomic community and urban signature OTUs both closely resembled sewage.

Sewage contains an integrated signal of human fecal organisms, but the bulk of the community is made up of organisms that are hypothesized to grow within the pipe infrastructure, either within biofilms or in sediments that settle to the bottom of pipes (VandeWalle et al., 2012; Newton et al., 2013). The composition of sewage communities is remarkably consistent over both time and geography (VandeWalle et al., 2012; Shanks et al., 2013; Newton et al., 2015), with the majority of variation stemming from differences in the non-fecal portion of the community (Newton et al., 2015). Just as sewage is a unique microbial ecosystem with distinct biogeographical patterns similar to those observed in natural ecosystems, stormwater exhibited spatial and temporal community composition cohesiveness, which is an indication that this built conveyance system is its own unique microbial ecosystem. Stormwater had comparatively high diversity in individual samples as well as the high total richness/diversity of the stormwater environment (pooled diversity). We suggest that stormwater is composed of both a consistent, ubiquitous community associated with drainage infrastructure and a highly variable community stemming from the variation in composition and magnitude of urban runoff sources such as soil.

Non-fecal taxa comprised the majority of the sewage signature taxa and represent organisms previously associated with pipes (Assanta et al., 2002; Bomo et al., 2004; Berry et al., 2006; Liu et al., 2014). Five abundant genera, *Acinetobacter, Arcobacter, Aeromonas, Pseudomonas*, and *Trichococcus*, were all present in stormwater samples; but only *Aeromonas, Acinetobacter*, and *Pseudomonas* were abundant. *Trichococcus* and *Arcobacter* were present in very low abundance in all stormwater samples, except for the one sewage-contaminated site, where they were highly abundant. Conversely, *Pseudomonas*, a taxon associated with both soil (Timmis, 2002) and pipes (Liu et al., 2014) had a higher relative abundance in stormwater compared to sewage. A recent study of drinking water pipes showed that loose sediments in the pipes, rather than biofilms, provide the majority of organisms that flux from the pipe environment (Liu et al., 2014); such sediments may also be important in stormwater and sanitary sewers for growth of our dominant urban signature bacteria.

The ratios of the dominant pipe organisms distinguished the sanitary sewage infrastructure signature from the stormwater infrastructure signature, suggesting that pipe environments select for a common group of organisms, while the particular input (stormwater versus sanitary sewage) determines the specific organism composition and their relative abundance. The OTU composition of *Acinetobacter* and *Pseudomonas* was more uniformly diverse in stormwater than what was found in sewage, which only contained two dominant members as defined by the MED OTUs (this study) or V6 pyrotag sequences (VandeWalle et al., 2012). *Aeromonas* also had two dominant OTUs that were found in stormwater and sewage, and several less abundant OTUs that were also found in similar proportions. *Arcobacter* and *Trichococcus* were fairly rare in stormwater, and only one dominant OTU was found for each of these in both sewage and stormwater. Many studies have addressed the effect of pipe materials on the growth of different bacteria (Chang et al., 2003; Yu et al., 2010), but here the differences in inputs (sanitary sewage versus runoff) to each system impact the relative abundance of these core pipe-associated organisms (Srinivasan et al., 2008). The OTUs in stormwater that are not present in sewage likely represent the runoff community, particularly organisms from soil.

### Urban signature in the aquatic environment

We were able to track the urban bacterial signature taxa and OTUs within those taxa into the aquatic environment by first establishing the natural communities from the lake, estuary, and rivers. These aquatic communities have core members, but receive fluxes of organisms from various sources including sewage, stormwater, animal feces, and soil (McLellan and Salmore, 2003; Salmore et al., 2006). The exchange of bacteria between urban sources and the aquatic environment is a unidirectional flux; stormwater and sewage do not receive direct inputs from the lake, rivers, or harbor. Thus, the observation of an urban-derived OTU in both locations means that either it has been seeded from stormwater or sewage, or that the partial 16S rRNA gene sequence alone cannot differentiate between distinct organisms. In our analysis the original source of urban-derived OTUs is unknown (e.g., soil runoff may first pass through stormwater collection systems, then eventually wash into the rivers, harbor and lake), but we consider them generally as originating in an urban source.

The harbor is a mixing zone of the three rivers and the lake, which is reflected in the structure of the microbial community found there (Mueller-Spitz et al., 2009). The majority of the bacteria in the harbor were of lake origin, but significant portions of the community derive uniquely from both rivers (>10%) and urban sources (1.5–15%, depending on rain volume). The high proportions of urban signature OTUs in all river samples demonstrate that the rivers are conduit for urban bacteria to the harbor. In this study, we found increases of urban signatures following rainfall, and pronounced increases following a CSO event. The percentage of urban signature bacteria during a CSO event was nearly tenfold that of baseflow conditions for the harbor site. The dominant bacteria from the CSO urban signature were also present in baseflow samples, but at a lower relative abundance, suggesting that chronic as well as acute impacts occur. Previous work on the presence and extent of human fecal pollution in the estuary (Newton et al., 2013) support our findings that greater impacts were observed with increasing severity of weather-related events. Events such as CSOs, which combine both of the urban sources, have acute impacts on the harbor community, and a significant presence up to 8 km offshore as the runoff plume mixes with the lake. However, long-term persistence of these organisms has also been observed (Newton et al., 2013).

### Fate and function of urban microbes

Urban rivers and stormwater serve as the primary route for transporting urban bacteria to Lake Michigan, with additional inputs from sporadic events such as CSOs. Individual stormwater samples are highly diverse, both relative to taxa diversity within a sample and among different stormwater samples, as drainage areas within urban population centers can support a wide range of functional uses. The majority of the urban signature bacteria were Gammaproteobacteria, which are rare members of “natural” pelagic lake communities both in general and specifically in Lake Michigan (Mueller-Spitz et al., 2009; Newton et al., 2011a). The metabolic capability of these organisms is diverse; and many of these organisms can survive in low-nutrient conditions (e.g., *Pseudomonas, Aeromonas*), but have the ability to take advantage of richer environments (Hazen et al., 1978; Zavarzin et al., 1991; Gasol et al., 2002; Timmis, 2002). Although there is significant delivery of urban bacteria to the Milwaukee harbor, they have yet to take a foothold and outcompete the natural bacteria that are better adapted to the estuarine environment. Thus the chronic imprint of these organisms is currently limited to those that persist in the environment rather than flourish, but further research is needed to determine if these organisms are active or senescent. Furthermore, while the effects of urban signature input are minimized by exchange with the massive volume of Lake Michigan, greater impacts would likely be observed in smaller inland lakes or river systems.

## Conclusions

Just as urbanization has affected available habitats for macroorganisms (Paul and Meyer, 2001; Grimm et al., 2008), increases in impervious ground cover and the presence of infrastructure change the diversity and composition of available habitats for microbial growth in urban areas. Stormwater and sewage pipe systems in particular provide niches that foster the growth of organisms that otherwise have a low relative abundance in the natural environment. Infrastructure- and soil-associated organisms make up the majority of the urban bacterial flux and can be found in adjacent aquatic environments, where they appear to maintain a small but persistent existence. Factors such as climate change and urban sprawl will only exacerbate the influence of urban-derived microorganisms on the surrounding environment. While these organisms may not be directly associated with human health risks, their impact on the local ecosystem function should be further considered, particularly in smaller systems that lack the dilution capacity of the Great Lakes.

## Supplementary Material

Table S1

Table S2

Table S3

## Figures and Tables

**Table 1:** Collection dates and rainfall intensities for study samples

Sample Type	Sample IDs	Sample Date	Weather
Lake	MH1, MH2, MH3	6/04/12	baseflow^a^
	DP1, DP2, DP3		
Rivers	KK1, MKE1, MNE1	7/28/11	rain
	KK2, MKE2, MNE2	10/14/11	antecedent rain^b^
Estuary/Harbor	Junction1, Gap1	6/04/12	baseflow
	Junction2, Gap2	8/23/11	<0.5 cm
	Junction3, Gap3	9/28/11	antecedent rain
	Junction4, Gap4	6/22/11	baseflow
Sewage	JI1, JI2, JI3	8/07/12, 4/04/12, 5/01/13	baseflow
	SS1, SS2	1/25/11, 4/11/11	baseflow
Stormwater	HC23	6/04/10	rain
	MN40	9/01/10	rain
	MN53	10/26/10	rain
	MN73	6/15/11	rain
	UC09, UC10	6/20/11	rain
	HAC13, HAC21, HAC22, HC33, HC43	7/22/11	rain
	HAC16	9/29/11	rain
	SWC01B	6/16/12	rain

a Baseflow conditions reflect no rain on the day of sampling and no rain 48 h prior to sampling

b Antecedent rain conditions reflect no rain on the day of sampling but rainfall >1 cm within 24 h of sampling.

doi: 10.12952/journal.elementa.000064.t001
