# Supplementary material for: Urban microbial ecology of a freshwater estuary of Lake Michigan
Source: Elementa (Wash D C). Author manuscript; Available in PMC 2016 Feb 8. (PMC4746012; doi:10.12952/journal.elementa.000064)
Supplement: Table S1 [file NIHMS719611-supplement-Table_S1.pdf]

Supplementary Table S1. Sample Metadata.
Sample metadata includes the Sample ID used in the paper, the sequencing ID that can be used to reference the raw sequence file in the NCBI Short Read Archive, the sample collection date, the type of environment from which the sample was collected, and the amount of rainfall or conditions when the sample was collected. Baseflow indicates no rain or significant antecedent rainfall. CSO = Combined sewerage overflow.

Site ID	Sequence ID (SRA)	Sample Date	Environment	Weather*
MH1	152_1_mile_06_04_12	6/4/12	Lake	baseflow
MH2	153_2_mile_06_04_12	6/4/12	Lake	baseflow
MH3	154_5_mile_06_04_12	6/4/12	Lake	baseflow
DP1	155_Doc_In_06_04_12	6/4/12	Lake	baseflow
DP2	156_Doc_Mid_06_04_12	6/4/12	Lake	baseflow
DP3	157_Doc_Out_06_04_12	6/4/12	Lake	baseflow
Junction4	50_Junction_6_22_11	6/22/11	Harbor	0.69, CSO
Junction2	52_Junction_8_23_11	8/23/11	Harbor	<0.5
Junction3	54_Junction_9_28_11	9/28/11	Harbor	antecedent rain
Junction1	150_Junction_06_04_12	6/4/12	Harbor	baseflow
Gap4	51_Gap_6_22_11	6/22/11	Harbor	0.69, CSO
Gap2	53_Gap_8_23_11	8/23/11	Harbor	<0.5
Gap3	55_Gap_9_28_11	9/28/11	Harbor	antecedent rain
Gap1	151_Gap_6_04_12	6/4/12	Harbor	baseflow
KK1	160_KK_River_07_28_11	7/28/11	River	4.2
KK2	161_KK_River_10_14_11	10/14/11	River	antecedent rain
MKE1	158_MKE_River_07_28_11	7/28/11	River	4.2
MKE2	162_MKE_River_10_14_11	10/14/11	River	antecedent rain
MNE1	159_MNE_River_07_28_11	7/28/11	River	4.2
MNE2	163_MNE_River_10_14_11	10/14/11	River	antecedent rain
JI1	164_JI_WWTP_Day1_08_07_12	8/7/12	Sewage	baseflow
JI2	49_JI_WWTP_4_4_12	4/4/12	Sewage	baseflow
JI3	199_Milwaukee JI	5/1/13	Sewage	baseflow
SS1	SS_WWTP_1_25_11_16s	1/25/11	Sewage	baseflow
SS2	SS_WWTP_4_11_11_16s	4/11/11	Sewage	baseflow
HC23	17_FMRHC23_6_4_10	6/4/10	Stormwater	0.46
MN40	23_FMRMN40_9_1_10	9/1/10	Stormwater	1.60
MN53	26_FMRMN53_10_26_10	10/26/10	Stormwater	1.45
SWC01B	27_SWC01B_6_16_12	6/16/12	Stormwater	0.03
MN73	29_FMRMN73_6_15_11	6/15/11	Stormwater	0.46
UC10	32_FMRUC10_6_20_11	6/20/11	Stormwater	1.14
HAC13	36_FMRHAC13_7_22_11	7/22/11	Stormwater	0.86
HAC21	37_FMRHAC21_7_22_11	7/22/11	Stormwater	0.86
HAC22	38_FMRHAC22_7_22_11	7/22/11	Stormwater	0.86

HC33	42_FMRHC33_7_22_11	7/22/11	Stormwater	0.86
HC43	43_FMRHC43_7_22_11	7/22/11	Stormwater	0.86
UC09	46_FMRUC09_6_20_11	6/20/11	Stormwater	1.14
HAC16	47_FMRHAC16_9_29_11	9/29/11	Stormwater	0.48

*Rainfall values are given in cm.
