# Supplementary material for: Urban microbial ecology of a freshwater estuary of Lake Michigan
Source: Elementa (Wash D C). Author manuscript; Available in PMC 2016 Feb 8. (PMC4746012; doi:10.12952/journal.elementa.000064)
Supplement: Table S2 [file NIHMS719611-supplement-Table_S2.pdf]

Supplementary Tables S2. Sample diversity analysis

Supplementary Table S2A. Hill Diversity (effective number of species) was calculated for four diversity orders (0, 1, 2 and infinity) that correspond to the common diversity indices Richness, Shannon Diversity, Inverse Simpson Diversity and Minimum Entropy.

	**Hill Diversity of Taxa by Sample**			
	**q=0**	**q=1**	**q=2**	**q=∞**
Lake DP1	557	22.4	11.4	4.4
Lake DP2	615	20.8	9.6	3.7
Lake DP3	575	22.2	10.7	4.1
Lake MH1	649	24.7	10.9	4.0
Lake MH2	666	23.1	10.1	3.7
Lake MH3	584	22.3	10.5	3.9
Harbor Gap1	705	32.8	16.6	5.7
Harbor Gap2	760	43.7	20.1	6.3
Harbor Gap3	1153	53.2	21.7	7.3
Harbor Gap4	1479	68.6	21.6	6.5
Harbor Junction1	936	45.0	20.5	7.6
Harbor Junction2	924	56.1	21.9	5.9
Harbor Junction3	1233	46.6	18.3	6.9
Harbor Junction4	1546	69.3	20.3	5.8
River KK1	1311	45.1	13.2	4.7
River KK2	940	44.2	21.8	8.3
River MKE1	1446	72.0	26.2	8.4
River MKE2	1148	32.9	11.0	3.8
River MNE1	1252	45.0	14.6	4.9
River MNE2	983	32.9	14.1	5.0
SW HAC13	1509	171.9	68.3	16.0
SW HAC16	1404	93.8	39.2	11.3
SW HAC21	1152	57.3	13.5	4.1
SW HAC22	1125	39.2	11.8	4.2
SW HC23	1461	113.1	38.6	10.4
SW HC33	1629	152.3	64.7	20.4
SW HC43	1217	50.1	21.8	9.3
SW MN40	1675	176.1	67.2	17.4
SW MN53	1132	39.9	13.5	4.5
SW MN73	1107	27.7	9.3	3.6
SW UC09	1429	112.0	40.7	10.9
SW UC10	1569	133.3	52.1	15.2
SWC01B	1061	31.9	10.8	3.9
Sewage JI1	1073	46.1	20.1	7.0

Sewage JI2	1149	31.3	11.9	5.0
Sewage JI3	1410	55.3	20.8	7.1
Sewage SS1	1029	50.6	18.4	5.7
Sewage SS2	1051	37.1	14.5	5.9

Supplementary Table S2B. Hill Diversity (effective species numbers) was calculated for four diversity orders (0, 1, 2 and infinity) that correspond to the common diversity indices Richness, Shannon Diversity, Inverse Simpson Diversity and Minimum Entropy. The *renyiaccum* function in R was used to calculate Hill numbers for each environment based on the average of all samples or all samples pooled.

	**Hill Diversity of Taxa by Environment**			
	**q=0**	**q=1**	**q=2**	**q=∞**
**Lake, average**	608.0	22.5	10.5	4.0
**Lake, pooled**	1062.0	23.1	10.6	4.0
**Harbor, average**	1101.9	52.3	20.2	6.5
**Harbor, pooled**	1970.0	60.3	23.0	7.7
**River, average**	1177.7	45.5	17.0	5.9
**River, pooled**	1869.0	55.5	18.3	5.4
**Stormwater, average**	1339.6	91.2	34.4	10.0
**Stormwater, pooled**	2567.0	148.8	50.0	12.3
**Sewage, average**	1141.2	44.0	17.1	6.2
**Sewage, pooled**	1830.0	52.5	20.9	9.0
