# Supplementary material for: Urban microbial ecology of a freshwater estuary of Lake Michigan
Source: Elementa (Wash D C). Author manuscript; Available in PMC 2016 Feb 8. (PMC4746012; doi:10.12952/journal.elementa.000064)
Supplement: Table S3 [file NIHMS719611-supplement-Table_S3.pdf]

Supplementary Table S3. LEfSE biomarker taxa results

The analysis using specific environmental classes included sewage, stormwater, river, harbor, and lake. The general classes were urban, which grouped sewage and stormwater and aquatic, which grouped river, harbor, and lake. The specific environment analysis produced significantly more biomarkers (530) than the general analysis (279). Most biomarker taxa identified in the general analysis agreed with the specific analysis (i.e., Urban biomarkers were also Stormwater or Sewage biomarkers and Aquatic biomarkers were also River, Harbor, or Lake biomarkers).

	Environment	
**Taxon**	**Specific**	**General**
Bacteria_Acidobacteria_Holophagae	Stormwater	Urban
Bacteria_Actinobacteria_Actinobacteria_Acidimicrobiales	Stormwater	Urban
Bacteria_Actinobacteria_Actinobacteria_Actinomycetales	Stormwater	Urban
Bacteria_Actinobacteria_Actinobacteria_Actinomycetales_Cellulomonadaceae	Stormwater	Urban
Bacteria_Actinobacteria_Actinobacteria_Actinomycetales_Cellulomonadaceae_Cellulomonas	Stormwater	Urban
Bacteria_Actinobacteria_Actinobacteria_Actinomycetales_Corynebacteriaceae_Corynebacterium_glutamicum	Stormwater	Urban
Bacteria_Actinobacteria_Actinobacteria_Actinomycetales_Geodermatophilaceae_Blastococcus	Stormwater	Urban
Bacteria_Actinobacteria_Actinobacteria_Actinomycetales_Geodermatophilaceae_Modestobacter	Stormwater	Urban
Bacteria_Actinobacteria_Actinobacteria_Actinomycetales_Kineosporiaceae_Kineococcus	Stormwater	Urban
Bacteria_Actinobacteria_Actinobacteria_Actinomycetales_Microbacteriaceae_Leucobacter	Stormwater	Urban
Bacteria_Actinobacteria_Actinobacteria_Actinomycetales_Microbacteriaceae_Microbacterium	Stormwater	Urban
Bacteria_Actinobacteria_Actinobacteria_Actinomycetales_Micrococcaceae_Arthrobacter	Stormwater	Urban
Bacteria_Actinobacteria_Actinobacteria_Actinomycetales_Nocardiaceae	Stormwater	Urban
Bacteria_Actinobacteria_Actinobacteria_Actinomycetales_Nocardiaceae_Nocardia	Stormwater	Urban
Bacteria_Actinobacteria_Actinobacteria_Actinomycetales_Nocardiaceae_Rhodococcus	Stormwater	Urban
Bacteria_Actinobacteria_Actinobacteria_Actinomycetales_Nocardiaceae_Williamsia	Stormwater	Urban
Bacteria_Actinobacteria_Actinobacteria_Actinomycetales_Nocardioidaceae_Marmoricola	Stormwater	Urban
Bacteria_Actinobacteria_Actinobacteria_Actinomycetales_Nocardioidaceae_Nocardioides	Stormwater	Urban
Bacteria_Actinobacteria_Actinobacteria_Actinomycetales_Propionibacteriaceae	Stormwater	Urban
Bacteria_Actinobacteria_Actinobacteria_Actinomycetales_Propionibacteriaceae_Friedmanniella	Stormwater	Urban
Bacteria_Actinobacteria_Actinobacteria_Actinomycetales_Propionibacteriaceae_Microlunatus	Stormwater	Urban

Bacteria_Actinobacteria_Actinobacteria_Actinomycetales_Streptomycetaceae_Streptomyces	Stormwater	Urban
Bacteria_Actinobacteria_Actinobacteria_Solirubrobacterales	Stormwater	Urban
Bacteria_Actinobacteria_Actinobacteria_Solirubrobacterales_Patulibacteraceae_Patulibacter	Stormwater	Urban
Bacteria_Bacteroidetes_Bacteroidia_Bacteroidales_Porphyromonadaceae	Stormwater	Urban
Bacteria_Bacteroidetes_Flavobacteria_Flavobacteriales_Flavobacteriaceae	Stormwater	Urban
Bacteria_Bacteroidetes_Flavobacteria_Flavobacteriales_Flavobacteriaceae_Chryseobacterium	Stormwater	Urban
Bacteria_Bacteroidetes_Flavobacteria_Flavobacteriales_Flavobacteriaceae_Elizabethkingia	Stormwater	Urban
Bacteria_Bacteroidetes_Flavobacteria_Flavobacteriales_Flavobacteriaceae_Myroides	Stormwater	Urban
Bacteria_Bacteroidetes_Sphingobacteria_Sphingobacteriales_Chitinophagaceae_Filimonas	Stormwater	Urban
Bacteria_Bacteroidetes_Sphingobacteria_Sphingobacteriales_Cytophagaceae_Dyadobacter	Stormwater	Urban
Bacteria_Bacteroidetes_Sphingobacteria_Sphingobacteriales_Cytophagaceae_Flexibacter	Stormwater	Urban
Bacteria_Bacteroidetes_Sphingobacteria_Sphingobacteriales_Cytophagaceae_Hymenobacter	Stormwater	Urban
Bacteria_Bacteroidetes_Sphingobacteria_Sphingobacteriales_Cytophagaceae_Spirosoma_rigui	Stormwater	Urban
Bacteria_Bacteroidetes_Sphingobacteria_Sphingobacteriales_Sphingobacteriaceae_Sphingobacteriaceae_Sphingobacterium	Stormwater	Urban
Bacteria_Chlamydiae_Chlamydiae_Chlamydiales_Parachlamydiaceae_Protochlamydia	Stormwater	Urban
Bacteria_Chloroflexi_Thermomicrobia_Sphaerobacterales_Sphaerobacteraceae_Sphaerobacter	Stormwater	Urban
Bacteria_Cyanobacteria	Stormwater	Urban
Bacteria_Cyanobacteria_Cyanobacteria	Stormwater	Urban
Bacteria_Cyanobacteria_Cyanobacteria_SubsectionII_SubgroupII_Chroococcidiopsis	Stormwater	Urban
Bacteria_Firmicutes_Bacilli_Bacillales_Bacillaceae_Bacillus	Stormwater	Urban
Bacteria_Firmicutes_Bacilli_Bacillales_Paenibacillaceae_Cohnella	Stormwater	Urban
Bacteria_Firmicutes_Bacilli_Bacillales_Paenibacillaceae_Paenibacillus	Stormwater	Urban
Bacteria_Firmicutes_Bacilli_Bacillales_Paenibacillaceae_Saccharibacillus	Stormwater	Urban
Bacteria_Firmicutes_Bacilli_Bacillales_Planococcaceae_Kurthia_Kurthia	Stormwater	Urban
Bacteria_Firmicutes_Bacilli_Bacillales_Unassigned_Exiguobacterium	Stormwater	Urban
Bacteria_Firmicutes_Bacilli_Lactobacillales_Leuconostocaceae_Leuconostoc	Stormwater	Urban
Bacteria_Firmicutes_Bacilli_Lactobacillales_Leuconostocaceae_Weissella	Stormwater	Urban
Bacteria_Firmicutes_Bacilli_Lactobacillales_Streptococcaceae_Streptococcus_castoreus	Stormwater	Urban

Bacteria_Firmicutes_Erysipelotrichi_Erysipelotrichales_Erysipelotrichaceae_Erysipelothrix	Stormwater	Urban
Bacteria_OD1	Stormwater	Urban
Bacteria_Planctomycetes	Stormwater	Urban
Bacteria_Planctomycetes_Phycisphaerae	Stormwater	Urban
Bacteria_Proteobacteria_Alphaproteobacteria	Stormwater	Urban
Bacteria_Proteobacteria_Alphaproteobacteria_Caulobacterales_Caulobacteraceae_Brevundimonas	Stormwater	Urban
Bacteria_Proteobacteria_Alphaproteobacteria_Rhizobiales_Aurantimonadaceae_Aurantimonas	Stormwater	Urban
Bacteria_Proteobacteria_Alphaproteobacteria_Rhizobiales_Bradyrhizobiaceae_Bosea	Stormwater	Urban
Bacteria_Proteobacteria_Alphaproteobacteria_Rhizobiales_Brucellaceae_Ochrobactrum	Stormwater	Urban
Bacteria_Proteobacteria_Alphaproteobacteria_Rhizobiales_Brucellaceae_Pseudochrobactrum	Stormwater	Urban
Bacteria_Proteobacteria_Alphaproteobacteria_Rhizobiales_Hyphomicrobiaceae_Devosia	Stormwater	Urban
Bacteria_Proteobacteria_Alphaproteobacteria_Rhizobiales_Phyllobacteriaceae_Mesorhizobium	Stormwater	Urban
Bacteria_Proteobacteria_Alphaproteobacteria_Rhizobiales_Rhizobiaceae_Rhizobium	Stormwater	Urban
Bacteria_Proteobacteria_Alphaproteobacteria_Rhodobacterales_Rhodobacteraceae_Amaricoccus	Stormwater	Urban
Bacteria_Proteobacteria_Alphaproteobacteria_Rhodobacterales_Rhodobacteraceae_Amaricoccus_tamworthensis	Stormwater	Urban
Bacteria_Proteobacteria_Alphaproteobacteria_Rhodobacterales_Rhodobacteraceae_Paracoccus	Stormwater	Urban
Bacteria_Proteobacteria_Alphaproteobacteria_Rhodobacterales_Rhodobacteraceae_Pseudorhodobacter	Stormwater	Urban
Bacteria_Proteobacteria_Alphaproteobacteria_Rhodobacterales_Rhodobacteraceae_Rubellimicrobium	Stormwater	Urban
Bacteria_Proteobacteria_Alphaproteobacteria_Rhodobacterales_Rhodobacteraceae_Rubrimonas	Stormwater	Urban
Bacteria_Proteobacteria_Alphaproteobacteria_Rhodospirillales	Stormwater	Urban
Bacteria_Proteobacteria_Alphaproteobacteria_Rhodospirillales_Acetobacteraceae_Acidiphilium	Stormwater	Urban
Bacteria_Proteobacteria_Alphaproteobacteria_Rhodospirillales_Acetobacteraceae_Roseomonas_vinacea	Stormwater	Urban
Bacteria_Proteobacteria_Alphaproteobacteria_Rhodospirillales_Acetobacteraceae_Teichococcus	Stormwater	Urban
Bacteria_Proteobacteria_Alphaproteobacteria_Rhodospirillales_Rhodospirillaceae	Stormwater	Urban
Bacteria_Proteobacteria_Alphaproteobacteria_Rhodospirillales_Rhodospirillaceae_Azospirillum	Stormwater	Urban
Bacteria_Proteobacteria_Alphaproteobacteria_Rhodospirillales_Rhodospirillaceae_Magnetospirillum	Stormwater	Urban
Bacteria_Proteobacteria_Alphaproteobacteria_Rhodospirillales_Rhodospirillaceae_Skermanella	Stormwater	Urban
Bacteria_Proteobacteria_Alphaproteobacteria_Rhodospirillales_Rhodospirillaceae_Thalassospira	Stormwater	Urban

Bacteria_Proteobacteria_Alphaproteobacteria_Sphingomonadales_Sphingomonadaceae_Sphingobium	Stormwater	Urban
Bacteria_Proteobacteria_Betaproteobacteria_Burkholderiales_Burkholderiaceae_Burkholderia	Stormwater	Urban
Bacteria_Proteobacteria_Betaproteobacteria_Burkholderiales_Comamonadaceae_Aquabacterium	Stormwater	Urban
Bacteria_Proteobacteria_Betaproteobacteria_Burkholderiales_Comamonadaceae_Giesbergeria	Stormwater	Urban
Bacteria_Proteobacteria_Betaproteobacteria_Burkholderiales_Comamonadaceae_Pelomonas	Stormwater	Urban
Bacteria_Proteobacteria_Betaproteobacteria_Burkholderiales_Comamonadaceae_Ramlibacter	Stormwater	Urban
Bacteria_Proteobacteria_Betaproteobacteria_Burkholderiales_Comamonadaceae_Roseateles	Stormwater	Urban
Bacteria_Proteobacteria_Betaproteobacteria_Burkholderiales_Oxalobacteraceae	Stormwater	Urban
Bacteria_Proteobacteria_Betaproteobacteria_Burkholderiales_Oxalobacteraceae_Duganella	Stormwater	Urban
Bacteria_Proteobacteria_Betaproteobacteria_Burkholderiales_Oxalobacteraceae_Herbaspirillum	Stormwater	Urban
Bacteria_Proteobacteria_Betaproteobacteria_Burkholderiales_Oxalobacteraceae_Herminiimonas	Stormwater	Urban
Bacteria_Proteobacteria_Betaproteobacteria_Burkholderiales_Oxalobacteraceae_Massilia	Stormwater	Urban
Bacteria_Proteobacteria_Betaproteobacteria_Burkholderiales_Oxalobacteraceae_Massilia_albidiflava	Stormwater	Urban
Bacteria_Proteobacteria_Betaproteobacteria_Burkholderiales_Oxalobacteraceae_Massilia_dura	Stormwater	Urban
Bacteria_Proteobacteria_Betaproteobacteria_Burkholderiales_Oxalobacteraceae_Oxalobacter	Stormwater	Urban
Bacteria_Proteobacteria_Betaproteobacteria_Neisseriales_Neisseriaceae_Aquaspirillum	Stormwater	Urban
Bacteria_Proteobacteria_Betaproteobacteria_Rhodocyclales_Rhodocyclaceae	Stormwater	Urban
Bacteria_Proteobacteria_Betaproteobacteria_Rhodocyclales_Rhodocyclaceae_Azoarcus	Stormwater	Urban
Bacteria_Proteobacteria_Betaproteobacteria_Rhodocyclales_Rhodocyclaceae_Azovibrio	Stormwater	Urban
Bacteria_Proteobacteria_Betaproteobacteria_Rhodocyclales_Rhodocyclaceae_Uliginosibacterium	Stormwater	Urban
Bacteria_Proteobacteria_Deltaproteobacteria_Bdellovibrionales_Bacteriovoraceae_Bacteriovorax	Stormwater	Urban
Bacteria_Proteobacteria_Deltaproteobacteria_Bdellovibrionales_Bdellovibrionaceae_Bdellovibrio	Stormwater	Urban
Bacteria_Proteobacteria_Deltaproteobacteria_Bdellovibrionales_Bdellovibrionaceae_Bdellovibrio_bacteriovorus	Stormwater	Urban
Bacteria_Proteobacteria_Deltaproteobacteria_Myxococcales_Cystobacteraceae	Stormwater	Urban
Bacteria_Proteobacteria_Deltaproteobacteria_Myxococcales_Haliangiaceae_Haliangium	Stormwater	Urban
Bacteria_Proteobacteria_Deltaproteobacteria_Myxococcales_Phaselicystidaceae_Phaselicystis	Stormwater	Urban
Bacteria_Proteobacteria_Deltaproteobacteria_Myxococcales_Polyangiaceae_Chondromyces	Stormwater	Urban
Bacteria_Proteobacteria_Deltaproteobacteria_Myxococcales_Polyangiaceae_Sorangium	Stormwater	Urban

Bacteria_Proteobacteria_Gammaproteobacteria	Stormwater	Urban
Bacteria_Proteobacteria_Gammaproteobacteria_Acidithiobacillales	Stormwater	Urban
Bacteria_Proteobacteria_Gammaproteobacteria_Alteromonadales_Idiomarinaceae_Idiomarina	Stormwater	Urban
Bacteria_Proteobacteria_Gammaproteobacteria_Alteromonadales_Shewanellaceae_Shewanella	Stormwater	Urban
Bacteria_Proteobacteria_Gammaproteobacteria_Chromatiales_Chromatiaceae_Alishewanella	Stormwater	Urban
Bacteria_Proteobacteria_Gammaproteobacteria_Enterobacteriales_Enterobacteriaceae	Stormwater	Urban
Bacteria_Proteobacteria_Gammaproteobacteria_Enterobacteriales_Enterobacteriaceae_Citrobacter	Stormwater	Urban
Bacteria_Proteobacteria_Gammaproteobacteria_Enterobacteriales_Enterobacteriaceae_Erwinia	Stormwater	Urban
Bacteria_Proteobacteria_Gammaproteobacteria_Enterobacteriales_Enterobacteriaceae_Erwinia_amylovora	Stormwater	Urban
Bacteria_Proteobacteria_Gammaproteobacteria_Enterobacteriales_Enterobacteriaceae_Pantoea	Stormwater	Urban
Bacteria_Proteobacteria_Gammaproteobacteria_Enterobacteriales_Enterobacteriaceae_Pantoea_agglomerans	Stormwater	Urban
Bacteria_Proteobacteria_Gammaproteobacteria_Oceanospirillales_Halomonadaceae_Halomonas	Stormwater	Urban
Bacteria_Proteobacteria_Gammaproteobacteria_Pseudomonadales_Moraxellaceae_Alkanindiges_illinoisensis	Stormwater	Urban
Bacteria_Proteobacteria_Gammaproteobacteria_Pseudomonadales_Moraxellaceae_Enhydrobacter	Stormwater	Urban
Bacteria_Proteobacteria_Gammaproteobacteria_Pseudomonadales_Pseudomonadaceae_Cellvibrio	Stormwater	Urban
Bacteria_Proteobacteria_Gammaproteobacteria_Pseudomonadales_Pseudomonadaceae_Cellvibrio_japonicus	Stormwater	Urban
Bacteria_Proteobacteria_Gammaproteobacteria_Pseudomonadales_Pseudomonadaceae_Pseudomonas	Stormwater	Urban
Bacteria_Proteobacteria_Gammaproteobacteria_Xanthomonadales_Xanthomonadaceae	Stormwater	Urban
Bacteria_Proteobacteria_Gammaproteobacteria_Xanthomonadales_Xanthomonadaceae_Stenotrophomonas	Stormwater	Urban
Bacteria_TM7	Stormwater	Urban
Bacteria_Actinobacteria_Actinobacteria_Actinomycetales_Actinomycetaceae_Actinomyces	Sewage	Urban
Bacteria_Actinobacteria_Actinobacteria_Actinomycetales_Corynebacteriaceae_Corynebacterium	Sewage	Urban
Bacteria_Actinobacteria_Actinobacteria_Actinomycetales_Dermacoccaceae_Dermacoccus	Sewage	Urban
Bacteria_Actinobacteria_Actinobacteria_Actinomycetales_Propionibacteriaceae_Tessaracoccus	Sewage	Urban
Bacteria_Actinobacteria_Actinobacteria_Actinomycetales_Sanguibacteraceae_Sanguibacter	Sewage	Urban
Bacteria_Actinobacteria_Actinobacteria_Bifidobacteriales_Bifidobacteriaceae_Bifidobacterium	Sewage	Urban
Bacteria_Actinobacteria_Actinobacteria_Coriobacteriales_Coriobacteriaceae_Collinsella	Sewage	Urban
Bacteria_Bacteroidetes_Bacteroidia_Bacteroidales	Sewage	Urban

Bacteria_Bacteroidetes_Bacteroidia_Bacteroidales_Bacteroidaceae_Bacteroides	Sewage	Urban
Bacteria_Bacteroidetes_Bacteroidia_Bacteroidales_Marinilabiaceae	Sewage	Urban
Bacteria_Bacteroidetes_Bacteroidia_Bacteroidales_Marinilabiaceae_Alkaliflexus	Sewage	Urban
Bacteria_Bacteroidetes_Bacteroidia_Bacteroidales_Porphyromonadaceae_Barnesiella	Sewage	Urban
Bacteria_Bacteroidetes_Bacteroidia_Bacteroidales_Porphyromonadaceae_Dysgonomonas	Sewage	Urban
Bacteria_Bacteroidetes_Bacteroidia_Bacteroidales_Porphyromonadaceae_Paludibacter	Sewage	Urban
Bacteria_Bacteroidetes_Bacteroidia_Bacteroidales_Porphyromonadaceae_Parabacteroides	Sewage	Urban
Bacteria_Bacteroidetes_Bacteroidia_Bacteroidales_Prevotellaceae_Prevotella	Sewage	Urban
Bacteria_Bacteroidetes_Bacteroidia_Bacteroidales_Rikenellaceae	Sewage	Urban
Bacteria_Bacteroidetes_Bacteroidia_Bacteroidales_Rikenellaceae_Alistipes	Sewage	Urban
Bacteria_Bacteroidetes_Flavobacteria_Flavobacteriales_Flavobacteriaceae_Cloacibacterium	Sewage	Urban
Bacteria_Bacteroidetes_Flavobacteria_Flavobacteriales_Flavobacteriaceae_Empedobacter	Sewage	Urban
Bacteria_Bacteroidetes_Flavobacteria_Flavobacteriales_Flavobacteriaceae_Lutibacter	Sewage	Urban
Bacteria_Firmicutes_Bacilli_Lactobacillales	Sewage	Urban
Bacteria_Firmicutes_Bacilli_Lactobacillales_Carnobacteriaceae_Trichococcus	Sewage	Urban
Bacteria_Firmicutes_Bacilli_Lactobacillales_Enterococcaceae_Enterococcus	Sewage	Urban
Bacteria_Firmicutes_Bacilli_Lactobacillales_Lactobacillaceae_Lactobacillus	Sewage	Urban
Bacteria_Firmicutes_Bacilli_Lactobacillales_Streptococcaceae_Lactococcus	Sewage	Urban
Bacteria_Firmicutes_Bacilli_Lactobacillales_Streptococcaceae_Streptococcus	Sewage	Urban
Bacteria_Firmicutes_Clostridia_Clostridiales_Clostridiaceae_Clostridium	Sewage	Urban
Bacteria_Firmicutes_Clostridia_Clostridiales_Eubacteriaceae_Acetobacterium	Sewage	Urban
Bacteria_Firmicutes_Clostridia_Clostridiales_Lachnospiraceae	Sewage	Urban
Bacteria_Firmicutes_Clostridia_Clostridiales_Lachnospiraceae_Blautia	Sewage	Urban
Bacteria_Firmicutes_Clostridia_Clostridiales_Lachnospiraceae_Coprococcus	Sewage	Urban
Bacteria_Firmicutes_Clostridia_Clostridiales_Lachnospiraceae_Dorea	Sewage	Urban
Bacteria_Firmicutes_Clostridia_Clostridiales_Lachnospiraceae_Roseburia	Sewage	Urban
Bacteria_Firmicutes_Clostridia_Clostridiales_Peptostreptococcaceae	Sewage	Urban
Bacteria_Firmicutes_Clostridia_Clostridiales_Peptostreptococcaceae_Fusibacter	Sewage	Urban

Bacteria_Firmicutes_Clostridia_Clostridiales_Ruminococcaceae	Sewage	Urban
Bacteria_Firmicutes_Clostridia_Clostridiales_Ruminococcaceae_Acetivibrio	Sewage	Urban
Bacteria_Firmicutes_Clostridia_Clostridiales_Ruminococcaceae_Oscillibacter	Sewage	Urban
Bacteria_Firmicutes_Clostridia_Clostridiales_Ruminococcaceae_Ruminococcus	Sewage	Urban
Bacteria_Firmicutes_Clostridia_Clostridiales_Ruminococcaceae_Subdoligranulum	Sewage	Urban
Bacteria_Firmicutes_Clostridia_Clostridiales_Unassigned_Anaerovorax	Sewage	Urban
Bacteria_Firmicutes_Clostridia_Clostridiales_Veillonellaceae	Sewage	Urban
Bacteria_Firmicutes_Clostridia_Clostridiales_Veillonellaceae_Anaerospora	Sewage	Urban
Bacteria_Firmicutes_Clostridia_Clostridiales_Veillonellaceae_Sporomusa_acidovorans	Sewage	Urban
Bacteria_Firmicutes_Clostridia_Clostridiales_Veillonellaceae_Veillonella	Sewage	Urban
Bacteria_Firmicutes_Clostridia_Clostridiales_Veillonellaceae_Zymophilus	Sewage	Urban
Bacteria_Firmicutes_Erysipelotrichi_Erysipelotrichales_Erysipelotrichaceae	Sewage	Urban
Bacteria_Firmicutes_Erysipelotrichi_Erysipelotrichales_Erysipelotrichaceae_Coprobacillus	Sewage	Urban
Bacteria_Fusobacteria_Fusobacteria_Fusobacteriales_Fusobacteriaceae_Fusobacterium	Sewage	Urban
Bacteria_Fusobacteria_Fusobacteria_Fusobacteriales_Leptotrichiaceae	Sewage	Urban
Bacteria_Proteobacteria_Betaproteobacteria	Sewage	Urban
Bacteria_Proteobacteria_Betaproteobacteria_Burkholderiales_Comamonadaceae_Acidovorax	Sewage	Urban
Bacteria_Proteobacteria_Betaproteobacteria_Burkholderiales_Comamonadaceae_Simplicispira	Sewage	Urban
Bacteria_Proteobacteria_Betaproteobacteria_Burkholderiales_Oxalobacteraceae_Janthinobacterium	Sewage	Urban
Bacteria_Proteobacteria_Betaproteobacteria_Nitrosomonadales_Gallionellaceae	Sewage	Urban
Bacteria_Proteobacteria_Betaproteobacteria_Rhodocyclales_Rhodocyclaceae_Dechloromonas	Sewage	Urban
Bacteria_Proteobacteria_Betaproteobacteria_Rhodocyclales_Rhodocyclaceae_Ferribacterium	Sewage	Urban
Bacteria_Proteobacteria_Betaproteobacteria_Rhodocyclales_Rhodocyclaceae_Propionivibrio	Sewage	Urban
Bacteria_Proteobacteria_Betaproteobacteria_Rhodocyclales_Rhodocyclaceae_Thauera	Sewage	Urban
Bacteria_Proteobacteria_Betaproteobacteria_Rhodocyclales_Rhodocyclaceae_Zoogloea	Sewage	Urban
Bacteria_Proteobacteria_Deltaproteobacteria_Desulfovibrionales_Desulfomicrobiaceae_Desulfomicrobium	Sewage	Urban
Bacteria_Proteobacteria_Deltaproteobacteria_Desulfovibrionales_Desulfovibrionaceae_Desulfovibrio	Sewage	Urban
Bacteria_Proteobacteria_Epsilonproteobacteria_Campylobacterales_Campylobacteraceae_Arcobacter	Sewage	Urban

Bacteria_Proteobacteria_Epsilonproteobacteria_Campylobacterales_Campylobacteraceae_Sulfurospirillum	Sewage	Urban
Bacteria_Proteobacteria_Epsilonproteobacteria_Campylobacterales_Helicobacteraceae	Sewage	Urban
Bacteria_Proteobacteria_Gammaproteobacteria_Aeromonadales_Aeromonadaceae_Aeromonas	Sewage	Urban
Bacteria_Proteobacteria_Gammaproteobacteria_Aeromonadales_Aeromonadaceae_Tolumonas	Sewage	Urban
Bacteria_Proteobacteria_Gammaproteobacteria_Enterobacteriales_Enterobacteriaceae_Klebsiella	Sewage	Urban
Bacteria_Proteobacteria_Gammaproteobacteria_Enterobacteriales_Enterobacteriaceae_Yersinia	Sewage	Urban
Bacteria_Proteobacteria_Gammaproteobacteria_Pseudomonadales_Moraxellaceae_Acinetobacter	Sewage	Urban
Bacteria_Proteobacteria_Gammaproteobacteria_Pseudomonadales_Moraxellaceae_Alkanindiges	Sewage	Urban
Bacteria_Proteobacteria_Gammaproteobacteria_Pseudomonadales_Moraxellaceae_Psychrobacter	Sewage	Urban
Bacteria_Tenericutes_Mollicutes	Sewage	Urban
Bacteria_Actinobacteria_Actinobacteria_Actinomycetales_Beutenbergiaceae_Beutenbergia_cavernae		Urban
Bacteria_Actinobacteria_Actinobacteria_Actinomycetales_Micrococcaceae_Arthrobacter_cumminsii		Urban
Bacteria_Firmicutes_Clostridia_Clostridiales_Veillonellaceae_Dendrosporobacter		Urban
Bacteria_Firmicutes_Clostridia_Clostridiales_Veillonellaceae_Veillonella_dispar		Urban
Bacteria_Proteobacteria_Deltaproteobacteria_Desulfobacterales_Desulfobulbaceae_Desulforhopalus_singaporensis		Urban
Bacteria_Proteobacteria_Betaproteobacteria_Burkholderiales_Comamonadaceae_Polaromonas	Sewage	Aquatic
Bacteria_Actinobacteria_Actinobacteria_Actinomycetales_Microbacteriaceae	River	Aquatic
Bacteria_Actinobacteria_Actinobacteria_Actinomycetales_Microbacteriaceae_Rhodoluna	River	Aquatic
Bacteria_Bacteroidetes_Sphingobacteria_Sphingobacteriales_Chitinophagaceae_Lacibacter_cauensis	River	Aquatic
Bacteria_Bacteroidetes_Sphingobacteria_Sphingobacteriales_Cytophagaceae_Arcicella	River	Aquatic
Bacteria_Bacteroidetes_Sphingobacteria_Sphingobacteriales_Cytophagaceae_Emticicia	River	Aquatic
Bacteria_Bacteroidetes_Sphingobacteria_Sphingobacteriales_Cytophagaceae_Flectobacillus_lacus	River	Aquatic
Bacteria_Cyanobacteria_Cyanobacteria_SubsectionIII_Unassigned_Planktothrix	River	Aquatic
Bacteria_Proteobacteria_Alphaproteobacteria_Rhizobiales_Unassigned_Nordella	River	Aquatic
Bacteria_Proteobacteria_Alphaproteobacteria_Rhodobacterales_Rhodobacteraceae_Rhodobacter	River	Aquatic
Bacteria_Proteobacteria_Betaproteobacteria_Burkholderiales_Alcaligenaceae_Derxia	River	Aquatic
Bacteria_Proteobacteria_Betaproteobacteria_Burkholderiales_Burkholderiaceae_Polynucleobacter_cosmopolitanus	River	Aquatic
Bacteria_Proteobacteria_Betaproteobacteria_Burkholderiales_Comamonadaceae_Rhodoferax	River	Aquatic

Bacteria_Proteobacteria_Betaproteobacteria_Burkholderiales_Oxalobacteraceae_Paucimonas	River	Aquatic
Bacteria_Proteobacteria_Betaproteobacteria_Methylophilales_Methylophilaceae_Methylotenera	River	Aquatic
Bacteria_Proteobacteria_Gammaproteobacteria_Methylococcales_Methylococcaceae	River	Aquatic
Bacteria_Proteobacteria_Gammaproteobacteria_Methylococcales_Methylococcaceae_Methylococcus_capsulatus	River	Aquatic
Bacteria_Proteobacteria_Gammaproteobacteria_Methylococcales_Methylococcaceae_Methylomicrobium	River	Aquatic
Bacteria_Actinobacteria_Actinobacteria_Acidimicrobiales_Acidimicrobiaceae	Lake	Aquatic
Bacteria_Actinobacteria_Actinobacteria_Actinomycetales_Sporichthyaceae	Lake	Aquatic
Bacteria_Bacteroidetes_Flavobacteria_Flavobacteriales_Cryomorphaceae_Fluviicola	Lake	Aquatic
Bacteria_Bacteroidetes_Flavobacteria_Flavobacteriales_Flavobacteriaceae_Flavobacterium	Lake	Aquatic
Bacteria_Bacteroidetes_Sphingobacteria_Sphingobacteriales_Chitinophagaceae	Lake	Aquatic
Bacteria_Bacteroidetes_Sphingobacteria_Sphingobacteriales_Chitinophagaceae_Ferruginibacter	Lake	Aquatic
Bacteria_Bacteroidetes_Sphingobacteria_Sphingobacteriales_Cyclobacteriaceae_Algoriphagus	Lake	Aquatic
Bacteria_Bacteroidetes_Sphingobacteria_Sphingobacteriales_Saprospiraceae_Lewinella_antarctica	Lake	Aquatic
Bacteria_Chloroflexi	Lake	Aquatic
Bacteria_Cyanobacteria_Cyanobacteria_SubsectionI_Unassigned	Lake	Aquatic
Bacteria_Cyanobacteria_Cyanobacteria_SubsectionI_Unassigned_Synechococcus	Lake	Aquatic
Bacteria_Planctomycetes_Phycisphaerae_Phycisphaerales_Phycisphaeraceae	Lake	Aquatic
Bacteria_Proteobacteria_Alphaproteobacteria_Rhizobiales_Beijerinckiaceae_Methylocella	Lake	Aquatic
Bacteria_Proteobacteria_Alphaproteobacteria_Rickettsiales_SAR11_Pelagibacter	Lake	Aquatic
Bacteria_Proteobacteria_Alphaproteobacteria_Sphingomonadales_Sphingomonadaceae_Sphingopyxis	Lake	Aquatic
Bacteria_Proteobacteria_Betaproteobacteria_Burkholderiales_Alcaligenaceae	Lake	Aquatic
Bacteria_Proteobacteria_Gammaproteobacteria_Methylococcales_Methylococcaceae_Methylomonas_fodinarum	Lake	Aquatic
Bacteria_Verrucomicrobia_Opitutae_Opitutales_Opitutaceae_Opitutus	Lake	Aquatic
Bacteria_Verrucomicrobia_Verrucomicrobiae_Verrucomicrobiales_Verrucomicrobiaceae_Acidimethylosilex	Lake	Aquatic
Bacteria_Verrucomicrobia_Verrucomicrobiae_Verrucomicrobiales_Verrucomicrobiaceae_Luteolibacter	Lake	Aquatic
Bacteria_Actinobacteria_Actinobacteria_Actinomycetales_Microbacteriaceae_Aquiluna	Harbor	Aquatic
Bacteria_Actinobacteria_Actinobacteria_Actinomycetales_Microbacteriaceae_Planktoluna	Harbor	Aquatic
Bacteria_Actinobacteria_Actinobacteria_Actinomycetales_Sporichthyaceae_Planktophila	Harbor	Aquatic

Bacteria_Bacteroidetes	Harbor	Aquatic
Bacteria_Bacteroidetes_Flavobacteria_Flavobacteriales	Harbor	Aquatic
Bacteria_Bacteroidetes_Flavobacteria_Flavobacteriales_Cryomorphaceae_Fluviicola_taffensis	Harbor	Aquatic
Bacteria_Bacteroidetes_Flavobacteria_Flavobacteriales_Cryomorphaceae_Owenweeksia	Harbor	Aquatic
Bacteria_Bacteroidetes_Sphingobacteria_Sphingobacteriales	Harbor	Aquatic
Bacteria_Bacteroidetes_Sphingobacteria_Sphingobacteriales_Chitinophagaceae_Sediminibacterium	Harbor	Aquatic
Bacteria_Bacteroidetes_Sphingobacteria_Sphingobacteriales_Cytophagaceae_Leadbetterella	Harbor	Aquatic
Bacteria_Bacteroidetes_Sphingobacteria_Sphingobacteriales_Saprospiraceae_Aquirestis	Harbor	Aquatic
Bacteria_Bacteroidetes_Sphingobacteria_Sphingobacteriales_Saprospiraceae_Saprospira	Harbor	Aquatic
Bacteria_Bacteroidetes_Sphingobacteria_Sphingobacteriales_Sphingobacteriaceae	Harbor	Aquatic
Bacteria_Cyanobacteria_Cyanobacteria_SubsectionIII_Unassigned	Harbor	Aquatic
Bacteria_Cyanobacteria_Cyanobacteria_SubsectionIII_Unassigned_Prochlorothrix	Harbor	Aquatic
Bacteria_Proteobacteria_Alphaproteobacteria_Rhizobiales_Beijerinckiaceae	Harbor	Aquatic
Bacteria_Proteobacteria_Alphaproteobacteria_Rhizobiales_Methylocystaceae_Methylocystis	Harbor	Aquatic
Bacteria_Proteobacteria_Betaproteobacteria_Burkholderiales_Burkholderiaceae_Polynucleobacter	Harbor	Aquatic
Bacteria_Proteobacteria_Betaproteobacteria_Burkholderiales_Comamonadaceae_Curvibacter	Harbor	Aquatic
Bacteria_Proteobacteria_Betaproteobacteria_Burkholderiales_Comamonadaceae_Hydrogenophaga	Harbor	Aquatic
Bacteria_Proteobacteria_Betaproteobacteria_Methylophilales_Methylophilaceae	Harbor	Aquatic
Bacteria_Proteobacteria_Betaproteobacteria_Rhodocyclales_Rhodocyclaceae_Methyloversatilis	Harbor	Aquatic
Bacteria_Proteobacteria_Deltaproteobacteria_Bdellovibrionales_Bdellovibrionaceae	Harbor	Aquatic
Bacteria_Proteobacteria_Gammaproteobacteria_Chromatiales_Ectothiorhodospiraceae_Thiorhodospira	Harbor	Aquatic
Bacteria_Proteobacteria_Gammaproteobacteria_Enterobacteriales_Enterobacteriaceae_Sodalis	Harbor	Aquatic
Bacteria_Proteobacteria_Gammaproteobacteria_Methylococcales_Methylococcaceae_Methylocaldum	Harbor	Aquatic
Bacteria_Proteobacteria_Gammaproteobacteria_Oceanospirillales_Oceanospirillaceae_Pseudospirillum	Harbor	Aquatic
Bacteria_Proteobacteria_Gammaproteobacteria_Xanthomonadales_Xanthomonadaceae_Arenimonas	Harbor	Aquatic
Bacteria_Verrucomicrobia_Opitutae_Puniceicoccales_Puniceicoccaceae	Harbor	Aquatic
Bacteria_Verrucomicrobia_Spartobacteria	Harbor	Aquatic
Bacteria_Verrucomicrobia_Verrucomicrobiae_Verrucomicrobiales_Subdivision3	Harbor	Aquatic

Bacteria_Verrucomicrobia_Verrucomicrobiae_Verrucomicrobiales_Verrucomicrobiaceae	Harbor	Aquatic
Bacteria_Verrucomicrobia_Verrucomicrobiae_Verrucomicrobiales_Verrucomicrobiaceae_Haloferula	Harbor	Aquatic
Bacteria_Verrucomicrobia_Verrucomicrobiae_Verrucomicrobiales_Verrucomicrobiaceae_Prosthecobacter	Harbor	Aquatic
Bacteria_Verrucomicrobia_Verrucomicrobiae_Verrucomicrobiales_Verrucomicrobiaceae_Prosthecobacter_debontii	Harbor	Aquatic
Bacteria_Cyanobacteria_Cyanobacteria_SubsectionIV		Aquatic
Bacteria_Proteobacteria_Betaproteobacteria_Burkholderiales_Burkholderiaceae		Aquatic
Bacteria	Stormwater	
Bacteria_Acidobacteria_Acidobacteria_Acidobacteriales_Acidobacteriaceae	Stormwater	
Bacteria_Acidobacteria_Acidobacteria_Acidobacteriales_Acidobacteriaceae_Chloroacidobacterium	Stormwater	
Bacteria_Actinobacteria_Actinobacteria_Acidimicrobiales_Iamiaceae_Iamia	Stormwater	
Bacteria_Actinobacteria_Actinobacteria_Actinomycetales_Brevibacteriaceae_Brevibacterium	Stormwater	
Bacteria_Actinobacteria_Actinobacteria_Actinomycetales_Cellulomonadaceae_Actinotalea	Stormwater	
Bacteria_Actinobacteria_Actinobacteria_Actinomycetales_Dietziaceae_Dietzia	Stormwater	
Bacteria_Actinobacteria_Actinobacteria_Actinomycetales_Geodermatophilaceae	Stormwater	
Bacteria_Actinobacteria_Actinobacteria_Actinomycetales_Intrasporangiaceae_Knoellia	Stormwater	
Bacteria_Actinobacteria_Actinobacteria_Actinomycetales_Microbacteriaceae_Rathayibacter	Stormwater	
Bacteria_Actinobacteria_Actinobacteria_Actinomycetales_Micrococcaceae_Kocuria	Stormwater	
Bacteria_Actinobacteria_Actinobacteria_Actinomycetales_Micromonosporaceae	Stormwater	
Bacteria_Actinobacteria_Actinobacteria_Actinomycetales_Micromonosporaceae_Actinoplanes	Stormwater	
Bacteria_Actinobacteria_Actinobacteria_Actinomycetales_Micromonosporaceae_Micromonospora	Stormwater	
Bacteria_Actinobacteria_Actinobacteria_Actinomycetales_Nocardioidaceae_Aeromicrobium	Stormwater	
Bacteria_Actinobacteria_Actinobacteria_Nitriliruptorales_Nitriliruptoraceae_Nitriliruptor	Stormwater	
Bacteria_Actinobacteria_Actinobacteria_Solirubrobacterales_Conexibacteraceae_Conexibacter	Stormwater	
Bacteria_Actinobacteria_Actinobacteria_Solirubrobacterales_Solirubrobacteraceae_Solirubrobacter	Stormwater	
Bacteria_Bacteroidetes_Bacteroidia_Bacteroidales_Porphyromonadaceae_Symbiothrix	Stormwater	
Bacteria_Bacteroidetes_Flavobacteria_Flavobacteriales_Flavobacteriaceae_Gillisia	Stormwater	
Bacteria_Bacteroidetes_Flavobacteria_Flavobacteriales_Flavobacteriaceae_Tenacibaculum	Stormwater	
Bacteria_Bacteroidetes_Flavobacteria_Flavobacteriales_Flavobacteriaceae_Wautersiella	Stormwater	

Bacteria_Bacteroidetes_Sphingobacteria_Sphingobacteriales_Chitinophagaceae_Chitinophaga	Stormwater
Bacteria_Bacteroidetes_Sphingobacteria_Sphingobacteriales_Chitinophagaceae_Flavisolibacter	Stormwater
Bacteria_Bacteroidetes_Sphingobacteria_Sphingobacteriales_Chitinophagaceae_Niastella	Stormwater
Bacteria_Bacteroidetes_Sphingobacteria_Sphingobacteriales_Chitinophagaceae_Segetibacter	Stormwater
Bacteria_Bacteroidetes_Sphingobacteria_Sphingobacteriales_Chitinophagaceae_Terrimonas	Stormwater
Bacteria_Bacteroidetes_Sphingobacteria_Sphingobacteriales_Cyclobacteriaceae	Stormwater
Bacteria_Bacteroidetes_Sphingobacteria_Sphingobacteriales_Cytophagaceae	Stormwater
Bacteria_Bacteroidetes_Sphingobacteria_Sphingobacteriales_Cytophagaceae_Adhaeribacter	Stormwater
Bacteria_Bacteroidetes_Sphingobacteria_Sphingobacteriales_Cytophagaceae_Spirosoma	Stormwater
Bacteria_Bacteroidetes_Sphingobacteria_Sphingobacteriales_Sphingobacteriaceae_Sphingobacteriaceae	Stormwater
Bacteria_Bacteroidetes_Sphingobacteria_Sphingobacteriales_Sphingobacteriaceae_Sphingobacteriaceae_Mucilaginibacter	Stormwater
Bacteria_Bacteroidetes_Sphingobacteria_Sphingobacteriales_Sphingobacteriaceae_Sphingobacteriaceae_Pedobacter	Stormwater
Bacteria_BRC1	Stormwater
Bacteria_Chlamydiae_Chlamydiae_Chlamydiales	Stormwater
Bacteria_Chlamydiae_Chlamydiae_Chlamydiales_Simkaniaceae_Rhabdochlamydia	Stormwater
Bacteria_Chloroflexi_Anaerolineae_Anaerolineales_Anaerolinaceae	Stormwater
Bacteria_Chloroflexi_Chloroflexi_Herpetosiphonales_Herpetosiphonaceae_Herpetosiphon_aurantiacus	Stormwater
Bacteria_Cyanobacteria_Cyanobacteria_SubsectionII_SubgroupII_Pleurocapsa	Stormwater
Bacteria_Cyanobacteria_Cyanobacteria_SubsectionIII	Stormwater
Bacteria_Cyanobacteria_Cyanobacteria_SubsectionIV_Unassigned_Nostoc	Stormwater
Bacteria_Deinococcus_Thermus_Deinococci_Deinococcales_Deinococcaceae_Deinococcus	Stormwater
Bacteria_Fibrobacteres_Fibrobacteria_Fibrobacterales_Fibrobacteraceae	Stormwater
Bacteria_Firmicutes_Bacilli_Bacillales_Bacillaceae_Terribacillus	Stormwater
Bacteria_Firmicutes_Bacilli_Bacillales_Planococcaceae	Stormwater
Bacteria_Firmicutes_Bacilli_Bacillales_Unassigned_Exiguobacterium_sibiricum	Stormwater
Bacteria_Firmicutes_Clostridia_Clostridiales	Stormwater
Bacteria_Firmicutes_Clostridia_Clostridiales_Lachnospiraceae_Anaerosporobacter	Stormwater
Bacteria_Gemmatimonadetes_Gemmatimonadetes	Stormwater

Bacteria_Gemmatimonadetes_Gemmatimonadetes_Gemmatimonadales_Gemmatimonadaceae_Gemmatimonas	Stormwater
Bacteria_Nitrospirae_Nitrospira_Nitrospirales_Nitrospiraceae_Nitrospira	Stormwater
Bacteria_OP10	Stormwater
Bacteria_OP3	Stormwater
Bacteria_Planctomycetes_Planctomycetacia_Planctomycetales_Planctomycetaceae	Stormwater
Bacteria_Proteobacteria_Alphaproteobacteria_Caulobacterales_Caulobacteraceae	Stormwater
Bacteria_Proteobacteria_Alphaproteobacteria_Caulobacterales_Caulobacteraceae_Asticcacaulis	Stormwater
Bacteria_Proteobacteria_Alphaproteobacteria_Caulobacterales_Caulobacteraceae_Phenylobacterium	Stormwater
Bacteria_Proteobacteria_Alphaproteobacteria_Caulobacterales_Hyphomonadaceae	Stormwater
Bacteria_Proteobacteria_Alphaproteobacteria_Rhizobiales_Bradyrhizobiaceae	Stormwater
Bacteria_Proteobacteria_Alphaproteobacteria_Rhizobiales_Bradyrhizobiaceae_Balneimonas	Stormwater
Bacteria_Proteobacteria_Alphaproteobacteria_Rhizobiales_Bradyrhizobiaceae_Bradyrhizobium	Stormwater
Bacteria_Proteobacteria_Alphaproteobacteria_Rhizobiales_Hyphomicrobiaceae	Stormwater
Bacteria_Proteobacteria_Alphaproteobacteria_Rhizobiales_Hyphomicrobiaceae_Prosthecomicrobium	Stormwater
Bacteria_Proteobacteria_Alphaproteobacteria_Rhizobiales_Methylobacteriaceae_Methylobacterium	Stormwater
Bacteria_Proteobacteria_Alphaproteobacteria_Rhizobiales_Methylocystaceae	Stormwater
Bacteria_Proteobacteria_Alphaproteobacteria_Rhizobiales_Rhodobiaceae_Rhodobium	Stormwater
Bacteria_Proteobacteria_Alphaproteobacteria_Rhizobiales_Xanthobacteraceae	Stormwater
Bacteria_Proteobacteria_Alphaproteobacteria_Rhizobiales_Xanthobacteraceae_Pseudolabrys	Stormwater
Bacteria_Proteobacteria_Alphaproteobacteria_Rhodobacterales_Rhodobacteraceae_Seohaeicola	Stormwater
Bacteria_Proteobacteria_Alphaproteobacteria_Rhodospirillales_Acetobacteraceae	Stormwater
Bacteria_Proteobacteria_Alphaproteobacteria_Rhodospirillales_Acetobacteraceae_Roseococcus	Stormwater
Bacteria_Proteobacteria_Alphaproteobacteria_Rhodospirillales_Acetobacteraceae_Roseomonas	Stormwater
Bacteria_Proteobacteria_Alphaproteobacteria_Rhodospirillales_Rhodospirillaceae_Defluviicoccus	Stormwater
Bacteria_Proteobacteria_Alphaproteobacteria_Rhodospirillales_Rhodospirillaceae_Insolitispirillum_peregrinum	Stormwater
Bacteria_Proteobacteria_Alphaproteobacteria_Rickettsiales	Stormwater
Bacteria_Proteobacteria_Alphaproteobacteria_Rickettsiales_Holosporaceae_Holospora	Stormwater
Bacteria_Proteobacteria_Alphaproteobacteria_Rickettsiales_Unassigned_Caedibacter	Stormwater

Bacteria_Proteobacteria_Alphaproteobacteria_Sphingomonadales_Erythrobacteraceae	Stormwater
Bacteria_Proteobacteria_Alphaproteobacteria_Sphingomonadales_Erythrobacteraceae_Altererythrobacter	Stormwater
Bacteria_Proteobacteria_Alphaproteobacteria_Sphingomonadales_Erythrobacteraceae_Erythrobacter	Stormwater
Bacteria_Proteobacteria_Alphaproteobacteria_Sphingomonadales_Sphingomonadaceae_Sphingomonas	Stormwater
Bacteria_Proteobacteria_Betaproteobacteria_Burkholderiales	Stormwater
Bacteria_Proteobacteria_Betaproteobacteria_Burkholderiales_Burkholderiaceae_Limnobacter	Stormwater
Bacteria_Proteobacteria_Betaproteobacteria_Burkholderiales_Comamonadaceae_Azohydromonas	Stormwater
Bacteria_Proteobacteria_Betaproteobacteria_Burkholderiales_Comamonadaceae_Leptothrix	Stormwater
Bacteria_Proteobacteria_Betaproteobacteria_Burkholderiales_Comamonadaceae_Malikia	Stormwater
Bacteria_Proteobacteria_Betaproteobacteria_Burkholderiales_Comamonadaceae_Ottowia	Stormwater
Bacteria_Proteobacteria_Betaproteobacteria_Burkholderiales_Oxalobacteraceae_Duganella_zoogloeoides	Stormwater
Bacteria_Proteobacteria_Betaproteobacteria_Burkholderiales_Oxalobacteraceae_Naxibacter	Stormwater
Bacteria_Proteobacteria_Betaproteobacteria_Burkholderiales_Oxalobacteraceae_Oxalicibacterium_horti	Stormwater
Bacteria_Proteobacteria_Betaproteobacteria_Burkholderiales_Oxalobacteraceae_Undibacterium	Stormwater
Bacteria_Proteobacteria_Betaproteobacteria_Hydrogenophilales_Hydrogenophilaceae_Thiobacillus	Stormwater
Bacteria_Proteobacteria_Betaproteobacteria_Methylophilales_Methylophilaceae_Methylophilus	Stormwater
Bacteria_Proteobacteria_Betaproteobacteria_Methylophilales_Methylophilaceae_Methylovorus	Stormwater
Bacteria_Proteobacteria_Betaproteobacteria_Neisseriales_Neisseriaceae	Stormwater
Bacteria_Proteobacteria_Betaproteobacteria_Nitrosomonadales_Nitrosomonadaceae	Stormwater
Bacteria_Proteobacteria_Betaproteobacteria_Rhodocyclales_Rhodocyclaceae_Azospira	Stormwater
Bacteria_Proteobacteria_Betaproteobacteria_Rhodocyclales_Rhodocyclaceae_Ferribacterium_limneticum	Stormwater
Bacteria_Proteobacteria_Deltaproteobacteria	Stormwater
Bacteria_Proteobacteria_Deltaproteobacteria_Bdellovibrionales_Bacteriovoraceae_Peredibacter	Stormwater
Bacteria_Proteobacteria_Deltaproteobacteria_Desulfuromonadales_Desulfuromonadaceae_Desulfuromonas	Stormwater
Bacteria_Proteobacteria_Deltaproteobacteria_Desulfuromonadales_Geobacteraceae_Geobacter	Stormwater
Bacteria_Proteobacteria_Deltaproteobacteria_Myxococcales	Stormwater
Bacteria_Proteobacteria_Deltaproteobacteria_Myxococcales_Cystobacteraceae_Anaeromyxobacter	Stormwater
Bacteria_Proteobacteria_Deltaproteobacteria_Myxococcales_Polyangiaceae	Stormwater

Bacteria_Proteobacteria_Gammaproteobacteria_Alteromonadales_Alteromonadaceae_Teredinibacter	Stormwater
Bacteria_Proteobacteria_Gammaproteobacteria_Alteromonadales_Idiomarinaceae	Stormwater
Bacteria_Proteobacteria_Gammaproteobacteria_Chromatiales_Chromatiaceae_Rheinheimera	Stormwater
Bacteria_Proteobacteria_Gammaproteobacteria_Chromatiales_Halothiobacillaceae_Halothiobacillus	Stormwater
Bacteria_Proteobacteria_Gammaproteobacteria_Enterobacteriales_Enterobacteriaceae_Brenneria_salicis	Stormwater
Bacteria_Proteobacteria_Gammaproteobacteria_Enterobacteriales_Enterobacteriaceae_Cronobacter	Stormwater
Bacteria_Proteobacteria_Gammaproteobacteria_Enterobacteriales_Enterobacteriaceae_Enterobacter_cowanii	Stormwater
Bacteria_Proteobacteria_Gammaproteobacteria_Enterobacteriales_Enterobacteriaceae_Pantoea_oleae	Stormwater
Bacteria_Proteobacteria_Gammaproteobacteria_Legionellales_Coxiellaceae_Aquicella	Stormwater
Bacteria_Proteobacteria_Gammaproteobacteria_Legionellales_Coxiellaceae_Coxiella	Stormwater
Bacteria_Proteobacteria_Gammaproteobacteria_Legionellales_Legionellaceae_Legionella	Stormwater
Bacteria_Proteobacteria_Gammaproteobacteria_Oceanospirillales_Oceanospirillaceae	Stormwater
Bacteria_Proteobacteria_Gammaproteobacteria_Pseudomonadales_Moraxellaceae	Stormwater
Bacteria_Proteobacteria_Gammaproteobacteria_Pseudomonadales_Moraxellaceae_Perlucidibaca	Stormwater
Bacteria_Proteobacteria_Gammaproteobacteria_Pseudomonadales_Pseudomonadaceae_Pseudomonas_cichorii	Stormwater
Bacteria_Proteobacteria_Gammaproteobacteria_Xanthomonadales_Xanthomonadaceae_Lysobacter	Stormwater
Bacteria_Proteobacteria_Gammaproteobacteria_Xanthomonadales_Xanthomonadaceae_Rhodanobacter	Stormwater
Bacteria_Proteobacteria_Gammaproteobacteria_Xanthomonadales_Xanthomonadaceae_Thermomonas	Stormwater
Bacteria_Spirochaetes_Spirochaetes_Spirochaetales_Spirochaetaceae_Spirochaeta	Stormwater
Bacteria_Spirochaetes_Spirochaetes_Spirochaetales_Spirochaetaceae_Treponema	Stormwater
Bacteria_TG_1	Stormwater
Bacteria_TM6	Stormwater
Bacteria_Verrucomicrobia_Spartobacteria_Chthoniobacter	Stormwater
Bacteria_Actinobacteria_Actinobacteria_Actinomycetales_Intrasporangiaceae_Oryzihumus	Sewage
Bacteria_Actinobacteria_Actinobacteria_Actinomycetales_Microbacteriaceae_Pseudoclavibacter	Sewage
Bacteria_Actinobacteria_Actinobacteria_Actinomycetales_Micrococcaceae_Rothia	Sewage
Bacteria_Actinobacteria_Actinobacteria_Actinomycetales_Mycobacteriaceae_Mycobacterium	Sewage
Bacteria_Actinobacteria_Actinobacteria_Actinomycetales_Promicromonosporaceae_Cellulosimicrobium	Sewage

Bacteria_Actinobacteria_Actinobacteria_Actinomycetales_Propionibacteriaceae_Micropruina	Sewage
Bacteria_Actinobacteria_Actinobacteria_Actinomycetales_Propionibacteriaceae_Propionicicella	Sewage
Bacteria_Actinobacteria_Actinobacteria_Actinomycetales_Tsukamurellaceae_Tsukamurella	Sewage
Bacteria_Actinobacteria_Actinobacteria_Coriobacteriales_Coriobacteriaceae_Atopobium	Sewage
Bacteria_Actinobacteria_Actinobacteria_Coriobacteriales_Coriobacteriaceae_Olsenella	Sewage
Bacteria_Bacteroidetes_Bacteroidia_Bacteroidales_Porphyromonadaceae_Butyricimonas	Sewage
Bacteria_Bacteroidetes_Bacteroidia_Bacteroidales_Porphyromonadaceae_Odoribacter	Sewage
Bacteria_Bacteroidetes_Bacteroidia_Bacteroidales_Prevotellaceae_Paraprevotella	Sewage
Bacteria_Bacteroidetes_Bacteroidia_Bacteroidales_Prevotellaceae_Xylanibacter	Sewage
Bacteria_Bacteroidetes_Flavobacteria_Flavobacteriales_Flavobacteriaceae_Mariniflexile	Sewage
Bacteria_Bacteroidetes_Sphingobacteria_Sphingobacteriales_Cytophagaceae_Flexibacter_litoralis	Sewage
Bacteria_Fibrobacteres_Fibrobacteria_Fibrobacterales_Fibrobacteraceae_Fibrobacter	Sewage
Bacteria_Firmicutes_Bacilli_Bacillales_Staphylococcaceae_Nosocomiicoccus	Sewage
Bacteria_Firmicutes_Bacilli_Lactobacillales_Aerococcaceae_Aerococcus	Sewage
Bacteria_Firmicutes_Bacilli_Lactobacillales_Carnobacteriaceae	Sewage
Bacteria_Firmicutes_Bacilli_Lactobacillales_Carnobacteriaceae_Trichococcus_flocculiformis	Sewage
Bacteria_Firmicutes_Bacilli_Lactobacillales_Streptococcaceae_Streptococcus_parauberis	Sewage
Bacteria_Firmicutes_Clostridia_Clostridiales_Clostridiaceae_Acidaminobacter	Sewage
Bacteria_Firmicutes_Clostridia_Clostridiales_Eubacteriaceae	Sewage
Bacteria_Firmicutes_Clostridia_Clostridiales_Lachnospiraceae_Anaerostipes	Sewage
Bacteria_Firmicutes_Clostridia_Clostridiales_Lachnospiraceae_Lachnospira	Sewage
Bacteria_Firmicutes_Clostridia_Clostridiales_Lachnospiraceae_Moryella	Sewage
Bacteria_Firmicutes_Clostridia_Clostridiales_Ruminococcaceae_Anaerotruncus	Sewage
Bacteria_Firmicutes_Clostridia_Clostridiales_Ruminococcaceae_Butyricicoccus	Sewage
Bacteria_Firmicutes_Clostridia_Clostridiales_Ruminococcaceae_Faecalibacterium	Sewage
Bacteria_Firmicutes_Clostridia_Clostridiales_Veillonellaceae_Anaerosinus	Sewage
Bacteria_Firmicutes_Clostridia_Clostridiales_Veillonellaceae_Dendrosporobacter_quercicolus	Sewage
Bacteria_Firmicutes_Clostridia_Clostridiales_Veillonellaceae_Dialister	Sewage

Bacteria_Firmicutes_Clostridia_Clostridiales_Veillonellaceae_Megamonas	Sewage
Bacteria_Firmicutes_Clostridia_Clostridiales_Veillonellaceae_Megasphaera	Sewage
Bacteria_Firmicutes_Clostridia_Clostridiales_Veillonellaceae_Mitsuokella	Sewage
Bacteria_Firmicutes_Clostridia_Clostridiales_Veillonellaceae_Phascolarctobacterium	Sewage
Bacteria_Firmicutes_Clostridia_Clostridiales_Veillonellaceae_Propionispora_hippei	Sewage
Bacteria_Firmicutes_Clostridia_Clostridiales_Veillonellaceae_Selenomonas	Sewage
Bacteria_Firmicutes_Clostridia_Clostridiales_Veillonellaceae_Thermosinus	Sewage
Bacteria_Firmicutes_Erysipelotrichi_Erysipelotrichales_Erysipelotrichaceae_Catenibacterium	Sewage
Bacteria_Fusobacteria_Fusobacteria_Fusobacteriales_Fusobacteriaceae_Cetobacterium	Sewage
Bacteria_Fusobacteria_Fusobacteria_Fusobacteriales_Leptotrichiaceae_Leptotrichia	Sewage
Bacteria_Fusobacteria_Fusobacteria_Fusobacteriales_Leptotrichiaceae_Sebaldella_termitidis	Sewage
Bacteria_Proteobacteria_Betaproteobacteria_Burkholderiales_Alcaligenaceae_Parasutterella	Sewage
Bacteria_Proteobacteria_Betaproteobacteria_Burkholderiales_Alcaligenaceae_Sutterella	Sewage
Bacteria_Proteobacteria_Betaproteobacteria_Burkholderiales_Comamonadaceae_Diaphorobacter	Sewage
Bacteria_Proteobacteria_Betaproteobacteria_Burkholderiales_Comamonadaceae_Sphaerotilus	Sewage
Bacteria_Proteobacteria_Betaproteobacteria_Neisseriales_Neisseriaceae_Alysiella_crassa	Sewage
Bacteria_Proteobacteria_Betaproteobacteria_Neisseriales_Neisseriaceae_Aquaspirillum_serpens	Sewage
Bacteria_Proteobacteria_Betaproteobacteria_Neisseriales_Neisseriaceae_Chromobacterium	Sewage
Bacteria_Proteobacteria_Betaproteobacteria_Neisseriales_Neisseriaceae_Formivibrio	Sewage
Bacteria_Proteobacteria_Betaproteobacteria_Neisseriales_Neisseriaceae_Laribacter_hongkongensis	Sewage
Bacteria_Proteobacteria_Betaproteobacteria_Neisseriales_Neisseriaceae_Neisseria	Sewage
Bacteria_Proteobacteria_Betaproteobacteria_Neisseriales_Neisseriaceae_Uruburuella	Sewage
Bacteria_Proteobacteria_Deltaproteobacteria_Desulfobacterales_Desulfobulbaceae_Desulfobacterium	Sewage
Bacteria_Proteobacteria_Deltaproteobacteria_Desulfobacterales_Desulfobulbaceae_Desulfobulbus	Sewage
Bacteria_Proteobacteria_Epsilonproteobacteria_Campylobacterales_Campylobacteraceae_Arcobacter_butzleri	Sewage
Bacteria_Proteobacteria_Epsilonproteobacteria_Campylobacterales_Campylobacteraceae_Sulfurospirillum_deleyianum	Sewage
Bacteria_Proteobacteria_Epsilonproteobacteria_Campylobacterales_Helicobacteraceae_Sulfurimonas	Sewage
Bacteria_Proteobacteria_Gammaproteobacteria_Alteromonadales_Alteromonadaceae_SAR92	Sewage

Bacteria_Proteobacteria_Gammaproteobacteria_Enterobacteriales_Enterobacteriaceae_Pectobacterium	Sewage
Bacteria_Proteobacteria_Gammaproteobacteria_Enterobacteriales_Enterobacteriaceae_Yersinia_bercovieri	Sewage
Bacteria_Proteobacteria_Gammaproteobacteria_Thiotrichales_Thiotrichaceae_Thiothrix	Sewage
Bacteria_Spirochaetes_Spirochaetes_Spirochaetales	Sewage
Bacteria_Verrucomicrobia_Verrucomicrobiae_Verrucomicrobiales_Verrucomicrobiaceae_Akkermansia	Sewage
Bacteria_Actinobacteria_Actinobacteria_Actinomycetales_Frankiaceae_Frankia	River
Bacteria_Actinobacteria_Actinobacteria_Actinomycetales_Nocardioidaceae_Actinopolymorpha	River
Bacteria_Bacteroidetes_Sphingobacteria_Sphingobacteriales_Cytophagaceae_Flectobacillus	River
Bacteria_Bacteroidetes_Sphingobacteria_Sphingobacteriales_Cytophagaceae_Runella	River
Bacteria_Cyanobacteria_Cyanobacteria_SubsectionI_Unassigned_Microcystis	River
Bacteria_Cyanobacteria_Cyanobacteria_SubsectionIV_Unassigned_Anabaena	River
Bacteria_Cyanobacteria_Cyanobacteria_SubsectionIV_Unassigned_Aphanizomenon	River
Bacteria_Proteobacteria_Alphaproteobacteria_Rhizobiales	River
Bacteria_Proteobacteria_Alphaproteobacteria_Rhodobacterales_Rhodobacteraceae	River
Bacteria_Proteobacteria_Alphaproteobacteria_Rhodobacterales_Rhodobacteraceae_Catellibacterium	River
Bacteria_Proteobacteria_Alphaproteobacteria_Sphingomonadales	River
Bacteria_Proteobacteria_Alphaproteobacteria_Sphingomonadales_Sphingomonadaceae_Novosphingobium	River
Bacteria_Proteobacteria_Alphaproteobacteria_Sphingomonadales_Sphingomonadaceae_Novosphingobium_lentum	River
Bacteria_Proteobacteria_Betaproteobacteria_Burkholderiales_Comamonadaceae	River
Bacteria_Proteobacteria_Betaproteobacteria_Burkholderiales_Comamonadaceae_Comamonas	River
Bacteria_Proteobacteria_Betaproteobacteria_Burkholderiales_Comamonadaceae_Delftia	River
Bacteria_Proteobacteria_Betaproteobacteria_Burkholderiales_Comamonadaceae_Ideonella	River
Bacteria_Proteobacteria_Betaproteobacteria_Burkholderiales_Comamonadaceae_Leptothrix_cholodnii	River
Bacteria_Proteobacteria_Betaproteobacteria_Burkholderiales_Comamonadaceae_Macromonas	River
Bacteria_Proteobacteria_Betaproteobacteria_Burkholderiales_Comamonadaceae_Mitsuaria_chitosanitabida	River
Bacteria_Proteobacteria_Betaproteobacteria_Burkholderiales_Comamonadaceae_Sphaerotilus_natans	River
Bacteria_Proteobacteria_Betaproteobacteria_Burkholderiales_Unassigned_Thiomonas	River
Bacteria_Proteobacteria_Betaproteobacteria_Neisseriales_Neisseriaceae_Vogesella	River

Bacteria_Proteobacteria_Betaproteobacteria_Rhodocyclales_Rhodocyclaceae_Denitratisoma	River
Bacteria_Proteobacteria_Deltaproteobacteria_Syntrophobacterales_Syntrophaceae_Smithella	River
Bacteria_Proteobacteria_Gammaproteobacteria_Aeromonadales_Aeromonadaceae_Tolumonas_auensis	River
Bacteria_Proteobacteria_Gammaproteobacteria_Methylococcales_Methylococcaceae_Methylomonas	River
Bacteria_Proteobacteria_Gammaproteobacteria_Oceanospirillales_Halomonadaceae	River
Bacteria_Cyanobacteria_Cyanobacteria_SubsectionIII_Unassigned_Leptolyngbya	Lake
Bacteria_Proteobacteria_Alphaproteobacteria_Caulobacterales_Caulobacteraceae_Caulobacter	Lake
Bacteria_Proteobacteria_Alphaproteobacteria_Rickettsiales_Rickettsiaceae	Lake
Bacteria_Proteobacteria_Alphaproteobacteria_Rickettsiales_Unassigned_Captivus	Lake
Bacteria_Proteobacteria_Betaproteobacteria_Burkholderiales_Alcaligenaceae_Achromobacter	Lake
Bacteria_Proteobacteria_Betaproteobacteria_Burkholderiales_Burkholderiaceae_Ralstonia	Lake
Bacteria_Proteobacteria_Betaproteobacteria_Burkholderiales_Comamonadaceae_Methylibium	Lake
Bacteria_Bacteroidetes_Sphingobacteria_Sphingobacteriales_Saprospiraceae_Haliscomenobacter	Harbor
Bacteria_Chlorobi_Chlorobia_Chlorobiales	Harbor
Bacteria_Cyanobacteria_Cyanobacteria_SubsectionI	Harbor
Bacteria_Lentisphaerae_Lentisphaeria	Harbor
Bacteria_Proteobacteria_Alphaproteobacteria_Caulobacterales_Hyphomonadaceae_Hirschia	Harbor
Bacteria_Proteobacteria_Alphaproteobacteria_Sphingomonadales_Sphingomonadaceae	Harbor
Bacteria_Proteobacteria_Alphaproteobacteria_Sphingomonadales_Sphingomonadaceae_Sandarakinorhabdus_limnophila	Harbor
Bacteria_Proteobacteria_Betaproteobacteria_Burkholderiales_Comamonadaceae_Alicycliphilus	Harbor
Bacteria_Proteobacteria_Betaproteobacteria_Neisseriales_Neisseriaceae_Leeia	Harbor
Bacteria_Proteobacteria_Deltaproteobacteria_Desulfuromonadales	Harbor
Bacteria_Proteobacteria_Gammaproteobacteria_Alteromonadales_Alteromonadaceae	Harbor
Bacteria_Proteobacteria_Gammaproteobacteria_Alteromonadales_Alteromonadaceae_Marinobacter	Harbor
Bacteria_Proteobacteria_Gammaproteobacteria_Alteromonadales_Alteromonadaceae_Unassigned_Haliea	Harbor
Bacteria_Proteobacteria_Gammaproteobacteria_Alteromonadales_Idiomarinaceae_Pseudidiomarina	Harbor
Bacteria_Proteobacteria_Gammaproteobacteria_Oceanospirillales	Harbor
Bacteria_Proteobacteria_Gammaproteobacteria_Xanthomonadales_Sinobacteraceae	Harbor

Bacteria_Proteobacteria_Gammaproteobacteria_Xanthomonadales_Sinobacteraceae_Nevskia	Harbor
Bacteria_Proteobacteria_Gammaproteobacteria_Xanthomonadales_Sinobacteraceae_Steroidobacter	Harbor
Bacteria_Verrucomicrobia_Opitutae	Harbor
Bacteria_Verrucomicrobia_Verrucomicrobiae_Verrucomicrobiales_Verrucomicrobiaceae_Prosthecobacter_vanneervenii	Harbor
Bacteria_Verrucomicrobia_Verrucomicrobiae_Verrucomicrobiales_Verrucomicrobiaceae_Verrucomicrobium	Harbor
